# Neuroprotective effect of standardized *Vitex pubescens* Vahl bark extract and its *n*-butanol fraction against scopolamine-induced cognitive impairment in Sprague-Dawley rats in relationship to its isolated phytochemicals

**DOI:** 10.3389/fphar.2026.1775571

**Published:** 2026-04-17

**Authors:** Safa Abdelbaset, Iriny M. Ayoub, Rasha A. Tawfiq, Taghreed A. Majrashi, Mahmoud A. El Hassab, Wagdy Eldehna, Dina M. El-kersh, Omayma A. Eldahshan

**Affiliations:** 1 Pharmacognosy Department, Faculty of Pharmacy, The British University in Egypt (BUE), Cairo, Egypt; 2 Pharmacognosy Department, Faculty of Pharmacy, Ain Shams University, Cairo, Egypt; 3 Drug Research and Development Group (DRD-G), The British University in Egypt (BUE), Cairo, Egypt; 4 Pharmacology Department, Faculty of Pharmacy, The British University in Egypt (BUE), Cairo, Egypt; 5 Department of Pharmacognosy, College of Pharmacy, King Khalid University, Abha, Saudi Arabia; 6 Department of Medicinal Chemistry, Faculty of Pharmacy, King Salman International University (KSIU), El Tor, Egypt; 7 Department of Pharmaceutical Chemistry, Faculty of Pharmacy, Kafrelsheikh University, Kafr ElShaikh, Egypt; 8 Center for Drug Discovery Research and Development, Ain Shams University, Cairo, Egypt

**Keywords:** acetylcholinesterase, Alzheimer’s, ELISA, ellman’s assay, scopolamine, Vitex pubescens bark

## Abstract

**Introduction:**

Alzheimer’s disease (AD), marked by gradual cognitive decline and memory deterioration, poses a major global health concern and ranks as the fourth leading contributor to disability-adjusted life years. Genus *Vitex* is one of the largest genera of the family Lamiaceae, with reported neuroprotective activity attributed to the abundance of diverse bioactive metabolites. The current study aimed to investigate the phytochemical constituents of the defatted methanol extract of *Vitex pubescens* bark as well as evaluate its neuroprotective activity.

**Methods:**

The neuroprotective assessment includes histopathological examination, behavior tests, and biochemical analysis, *viz, in vivo* acetylcholinesterase (AChE) concentration *via* enzyme-linked immunosorbent assay (ELISA) and AChE inhibitory activity using Ellman’s microplate assay.

**Results:**

The phytochemical investigations revealed the isolation of six compounds, *viz*. betulinic acid, vanillic acid, *p*-hydroxybenzoic acid, orientin-2´ˊ-*O-α*-L-rhamnoside, isoorientin, and orientin. Histopathological examination indicated notable hippocampal recovery in the *n*-BuOH and orientin groups, exceeding improvements observed with other treated groups with defatted methanol extract.

**Discussion:**

The neuroprotective evaluation using behavioral and biochemical analyses demonstrated dose-dependent neuroprotective effects of the defatted methanol extract, with the *n*-BuOH fraction showing the strongest anti-dementia activity, reflected by enhanced AChE inhibition and improved neuronal integrity.

**Conclusion:**

The findings of this study reveal the potential of the defatted methanolic extract and *n*-buOH fraction of *Vitex pubescens* bark as a natural candidate for managing the progression of Alzheimer’s disease.

## 1 Introduction

Memory is a complex cognitive process that involves encoding, storing, and retrieving information (Zlotnik and Vansintjan, 2019). It plays a fundamental role in our daily lives, learning, and decision-making (Widmann et al., 2012). Memory deterioration is a hallmark feature of Alzheimer’s (AD) disease, which significantly extends to daily activities and the individual’s ability to perform tasks and recall information (Knopman et al., 2021). AD is considered the fourth leading cause of disability-adjusted life-years (DALYs), which induces an extraordinary economic burden (Gustavsson et al., 2023). AD is a neurodegenerative disease that is characterized by progressive impairment in cognitive functions, which subsequently leads to memory loss (Hampel et al., 2018). The incidence of AD is commonly noticed in elderly populations over 65 years, accounting for more than 25 million diagnosed cases worldwide, with an estimated 5 million new cases annually (Qiu et al., 2009).

The pathogenesis of Alzheimer’s disease has been explained through various mechanisms. The cholinergic hypothesis describes the degeneration of cholinergic neurons, decreasing acetylcholine (Ach) levels, and overactivity of the acetylcholinesterase (AchE) enzyme (Hampel et al., 2018; McGirr et al., 2020). Additionally, the deposition of senile neuritic plaques and neurofibrillary tangles induces neuronal dysfunction, neuronal cell death, and subsequently cognitive impairment. Oxidative stress and neuroinflammation are also considered major triggering factors, as they promote the accumulation of amyloid plaques, induce cytotoxicity, and cause neuronal deficits (Hosseinkhani et al., 2017).

Scopolamine (sco) is a standard drug used to induce dementia in a rat model *via* the blockage of muscarinic receptors because it is structurally similar to the Ach neurotransmitter, which competes with it on cholinergic receptors, causing cholinergic impairments (Kim et al., 2020). The approved conventional therapy by the Food and Drug Administration (FDA) for the treatment of AD is AchE inhibitors, *viz*., donepezil, rivastigmine, and galantamine. These drugs exhibit modest efficacy in managing AD symptoms without restoring the normal function of the neuron or providing a cure (Hansen et al., 2008). There are some undesirable side effects, such as hepatotoxicity, gastrointestinal upsets, and muscle cramps (Li et al., 2018). Interestingly, searching for natural entities with neuroprotective potential has been a significant area of interest for many researchers. Natural products provide a multitargeted approach that mainly exerts its neuroprotective action *via* exhibiting antioxidant and anti-inflammatory effects as well as inhibition of AchE.

Medicinal plants represent a valuable resource of nature’s healing abilities, intricately linked to human history and traditional healthcare practices. Ranging from ancient herbal treatments to contemporary pharmaceutical innovations, these plants hold considerable importance in both medicine and scientific exploration. Plants have been positioned as a primary treasure of bioactive compounds with promising medicinal and nutritional characteristics (Mostafa et al., 2018; Gamal El-Din et al., 2018).

*Vitex pubescens* Vahl (*Vitex pinnata* Linn) is a medicinal plant of the family Lamiaceae, widely scattered in territorial regions of Asia, including Indonesia, India, Malaysia, the Philippines, and Sri Lanka. The Malaysian *Vitex pubescens,* commonly known as “Halban,” was traditionally utilized in the treatment of various ailments (Anwar et al., 2019). The leaves were used as antipyretics, analgesics, and antifungals, as well as to relieve inflammation and heal gastric ulcers (Al-Akwaa et al., 2020; Saeed AL-Wajeeh et al., 2016). The bark was used for wound healing and in the treatment of stomachaches (Anwar et al., 2015). *V. pubescens* leaves were reported to exhibit various pharmacological activities, including gastroprotective, estrogenic, antihyperlipidemic, antihypertensive, antioxidant, antibacterial, and wound healing activities (Al-Akwaa et al., 2020; Saeed AL-Wajeeh et al., 2016; Promprom et al., 2020; Thenmozhi and Subasini, 2016). Furthermore, the bark was evaluated only for anti-leukemic and anti-tuberculosis activities (Anwar et al., 2019; Abdelbaset et al., 2023). Insufficient pharmacological investigations of the bark of *V. pubescens* have prompted the need for further exploration and research. Hence, this present study aimed to assess the neuroprotective activity of *V. pubescens* bark methanol extract and *n*-butanol (*n*-BuOH) fraction as well as phytochemical investigation in order to isolate the pure active compounds.

## 2 Materials and methods

### 2.1 Plant material

V. pubescens Vahl bark was collected from a native local plantation located in Orang Asali, Gerik, Perak, Malaysia. The plant was authenticated and purchased in December 2019 from ETHNO Resources Sdn. Bhd. (846944-K) herbal company, Selangor, Malaysia.^1^ A voucher specimen (PHG-P-VP-302) has been kept in the Pharmacognosy Department, Faculty of Pharmacy, Ain Shams University, Cairo, Egypt.

### 2.2 General solvents

The solvents, including *n*-hexane, dichloromethane (DCM), ethyl acetate (EtOAc), *n*-BuOH, and methanol, were purchased from Fisher Scientific, Loughborough, United Kingdom. Deuterated dimethyl sulphoxide (DMSO) and deuterated methanol (CD_3_OD) were used for ^1^H and ^13^C NMR measurements. Also, tetramethyl silane (TMS) was used as an internal standard, purchased from Deutero, Kastellaun, Germany.

Stationary phases used for fractionation, isolation, and purification, *viz*. silica gel for vacuum liquid chromatography (VLC), silica gel 60 for normal phase chromatography (70–230 mesh), (200–400mesh), (>400 mesh), and sephadex LH-20, were purchased from Sigma-Aldrich, St. Louis, United States. Diaion HP-20 and Polyamide-6 for column chromatography were obtained from Fluka, Steinheim, Germany.

### 2.3 Extraction and fractionation of *V. pubescens* bark

In total, 4 kg of *V. pubescens* bark powder was defatted using *n*-hexane at room temperature by the maceration method till exhaustion (3 × 10 L) to yield 18.7 g of *n*-hexane extract. The defatted *V. pubescens* powder was extracted with absolute methanol using the maceration method till exhaustion (7x15 L). Methanol was evaporated *in vacuo* to obtain 185 g (4.6% *w/w*) of the dried defatted methanol extract (VT), and then the extract was kept for successive liquid–liquid fractionation. The dried methanol extract (180 g) was suspended in the least amount of distilled water and subjected to liquid-liquid fractionation using solvents with increasing polarity, *viz*., ***n*-hexane** (4 × 1 L), followed by **DCM** (6 x 1L), **EtOAc** (3 × 1 L), and ***n*-BuOH** saturated with water (10 × 1 L). The solvent, in each case, was evaporated *in vacuo*. The dried solvent-free fractions were then weighed and calculated as %yield (weight of fraction (g)/weight of total extract (g) X100) as listed in Table 1 and kept at −20 °C for further chemical investigations (Brusotti et al., 2014).

**TABLE 1:** The weight and % yield of different fractions of *V. pubescens* defatted methanol extract.

Fraction	Weight (g)	Yield (%)
*n*-Hexane	10	5.6
DCM	21.1	11.7
EtOAc	5.5	3
*n*-BuOH	110	61.1
Mother liquor	20.7	11.5

### 2.4 Phytochemical investigation of the DCM fraction of *V. pubescens* bark

A total weight of 15 g of DCM fraction was subjected to fractionation using VLC (7.5 cm x13.5 cm) filled with silica gel for TLC (300 g), and the fraction was loaded using the dry method (Faisal et al., 2022; Abdelbaset et al., 2024). Elution was performed using a mixture of solvents, *viz*., *n*-hexane, DCM, EtOAc, and methanol in increasing polarity. Eluted subfractions (200 mL, each) were collected and monitored by thin-layer chromatography (TLC) plates using a solvent system (DCM: MeOH) (90:10). TLCs were visualized under UV lamp (254 nm and 365 nm) as well as using *p*-anisaldehyde spraying reagent. Similar subfractions were separately combined and weighed (yielded 7 subfractions), *viz*., **subfraction D-I** (780 mg), **subfraction D-II** (938 mg), **subfraction D-III** (2.23 g), **subfraction D-IV** (2 g), **subfraction D-V** (647 mg), **subfraction D-VI** (4.3 g), and **subfraction D-VII** (4 g). Three subfractions, **subfraction D-I** (780 mg; eluted from *n*-hexane: DCM 80:20 to 20:80 by increasing polarity with 10%), **subfraction D-II** (938 mg; eluted from *n*-hexane: DCM) 10:90 till 100% DCM by increasing polarity with 10%), and **subfraction D-III** (2.23 g; eluted from DCM: EtOAc 98:2 till 90:10 by increasing polarity with 2%) were purified using silica gel column chromatography to yield three pure compounds. **Compound V1** (29 mg) was isolated from **subfraction D-I** using mobile phase *n*-hexane: EtOAc (85 15). It appeared as a violet spot upon spraying with *p*-anisaldehyde reagent. **Compound V2** (68 mg) was isolated from **subfraction D-II** using mobile phase *n*-hexane: EtOAc (75:25) then further purified using DCM: MeOH (98:2). **Compound V3** (124 mg) was isolated from **subfraction D-III** using mobile phase *n*-hexane: EtOAc (85:15) then further purified using DCM: MeOH (98:2). The three pure **compounds (V1-3)** were subjected to spectroscopic analysis for structure elucidation.

### 2.5 Phytochemical investigation of the *n*-BuOH fraction of *V. pubescens* bark

A total weight of 85 g of the *n*-BuOH fraction (VB) was subjected to fractionation using Diaion HP-20 (225 g), applying column chromatography (44 cm × 5cm).

The fraction was loaded using the wet method, utilizing a mixture of water and MeOH in increasing polarity. Eluted subfractions (150 mL, each) were collected and monitored by TLC plates using the mobile phase (DCM: MeOH: formic acid) (80:20:2dps). TLC was visualized under UV lamp (254 nm and 365 nm) as well as using *p*-anisaldehyde spraying reagent. Similar subfractions were separately combined and weighed (yielding 8 subfractions), *viz*., **subfraction B-I** (10 g), **subfraction B-II** (29.31 g), **subfraction B-III** (2.03 g), **subfraction B-IV** (10.35 g), **subfraction B-V** (5.6 g), **subfraction B-VI** (2.09 g), **subfraction B-VII** (5.2 g), and **subfraction B-VIII** (20.42 g). The promising subfractions, **subfraction B-II** (29.31 g, eluted with 100% H_2_O) and **subfraction B-IV** (10.35 g, eluted using MeOH: H_2_O 50:50), were purified using polyamide 6 followed by Sephadex LH-20 column chromatography to yield three pure compounds: **compounds V4-6. Compound V4** (627 mg) was isolated from **subfraction B-II** using polyamide 6 as a stationary phase and eluted with mobile phase H_2_O: MeOH (75: 25); **compound V5** (75 mg) was isolated from **subfraction B-II** using polyamide 6 as stationary phase, eluted with mobile phase 100% MeOH then furtherly purified using column chromatography with sephadex LH-20, and eluted using mobile phase MeOH: H_2_O (95:5); and **compound V6** (913 mg) was isolated from **subfraction B-IV** using polyamide 6 as stationary phase, eluted with mobile phase 100% MeOH then furtherly purified using column chromatography with sephadex LH-20, and eluted using mobile phase 100% MeOH. The three pure **compounds V 4–6** were subjected to spectroscopic and spectrometric analyses for structure elucidation.

### 2.6 Nuclear magnetic resonance (NMR) spectroscopic analysis

^1^H and ^13^C-NMR analyses were performed using a Bruker Ascend 400/R spectrometer (Bruker Avance III, Fallanden, Switzerland) at operating frequencies of 400 and 100 MHz, respectively, at the Center of Drug Discovery Research and Development, Faculty of Pharmacy, Ain Shams University. Spectra were recorded at 25 °C; *δ* ppm with reference to TMS as an internal standard, and the chemical shift values were expressed in *δ* ppm. The samples were dissolved in deuterated solvents (DMSO-d_6_, CD_3_OD, Sigma Aldrich, Germany) and transferred into 3 mm NMR tubes (Bruker) (Youssef et al., 2017).

### 2.7 Ultra-performance liquid chromatography-photodiode array (UPLC-PDA)

#### 2.7.1 Preparation of stock and working solutions

The samples of the total methanol extract, *n*-BuOH fraction, and the major isolated flavonoids orientin, isoorientin, and orientin-2´ˊ-*O*-*α*-L-rhamnoside were prepared and utilized for qualitative and quantitative analysis. For qualitative analysis, the sample stock solutions were accurately prepared with a concentration of 1.0 mg/mL for each sample. For quantitative analysis, the calibration set of the tested samples (total methanol extract, *n*-BuOH fraction, and the isolated compounds orientin, isoorientin, and orientin-2´ˊ-*O*-*α*-L-rhamnoside) was prepared to obtain final concentrations of 5, 10, 50, 100, and 200 μg/mL. Subsequently, the prepared standards were injected into the UPLC-PDA, and the calibration curve of each component was constructed.

#### 2.7.2 Chromatographic conditions

The UPLC analysis was carried out using a Thermo Fisher Dionex UltiMate 3000 UPLC system with a photodiode array (PDA) detector, using a Hypersil Gold ^TM^ C_18_ column (250 × 4.6 mm and 3 μm particle size) as the stationary phase. The mobile phase was composed of two solvents: A [0.1% ortho-phosphoric acid in water (*v/v*) with pH adjusted to 3.5], and solvent B (acetonitrile). Gradient elution was performed as illustrated in Table 2 with a total run time of 60 min. The flow rate was 0.7 mL/min, and the injection volume was 20 µL. The column oven temperature was set at 25 °C, and the UV detector was adjusted to 340 nm (Abdelwahed et al., 2023).

**TABLE 2:** Time program for gradient elution using solvent (**A**) (0.1% ortho-phosphoric acid in water (*v/v*) with pH adjusted to 3.5) and solvent (**B**) (Acetonitrile).

Time (min)	%Solvent	
	A	B
0.00	95	5
3.00	95	5
25.00	85	15
53.00	65	35
60.00	90	5

### 2.8 Biological study

#### 2.8.1 Animals

A total of 41 male Sprague-Dawley rats weighing between 150 and 200 g were obtained from the animal house of the Faculty of Pharmacy, The British University in Egypt, Cairo, Egypt. The rats were housed under standard laboratory conditions at a temperature of 25 °C ± 2 °C and relative humidity of 55%–60% with free access to water and food. Handling, as well as the study procedures, were approved by the ethical committee of the Faculty of Pharmacy, The British University in Egypt, Cairo, Egypt (EX-2221), December 2022. Animals were divided into six groups, and each group of three rats was housed in the same cage for 7 days before starting the experiment for acclimatization.

#### 2.8.2 Animal experimental protocol

The rats were allocated randomly into six groups, each comprising six to seven rats. The animals were divided as follows.

- **Group I** (n = 6): received normal saline, *p.o.,* for 10 days (normal control group).
- **Group II** (n = 7): received scopolamine 1 mg/kg intraperitoneal (*i.p.*) for 10 days (Sco gp., served as negative control group).
- **Group III** (n = 7): received scopolamine 1 mg/kg, *i.p.*, then VT (150 mg/kg, *p.o.*) for 10 days (E150 gp.).
- **Group IV** (n = 7): received scopolamine 1 mg/kg, *i.p.*, then VT (300 mg/kg, *p.o.*) for 10 days (E300 gp.).
- **Group V** (n = 7): received scopolamine 1 mg/kg, *i.p.*, then VB (50 mg/kg, *p.o.*) for 10 days (VB gp).
- **Group VI** (n = 7): received scopolamine 1 mg/kg, *i.p.*, then orientin (10 mg/kg, *p.o.*) for 10 days (Ori group served as positive control group).

The experimental protocol was designed according to (El-Ganainy et al., 2021) through which scopolamine was injected (*i.p.*) with a dose of (1 mg/kg) (Rahiman et al., 2015) 1 hour before the treatment protocol with VT (150 and 300 mg/kg/day), VB (50 mg/kg/day) and orientin (10 mg/kg/day) as showed in Groups III, IV, V and VI; respectively through oral route (*p.o.*) using an intragastric tube.

These treatments were administered to rats as mentioned for 10 successive days. On the 8th, 9th, and 10th days, rats received scopolamine 30 min before the behavior tests to evaluate their memory and learning status. At the end of day 10, the rats were euthanized using the carbon dioxide (CO_2_) inhalation method, and the brain tissues were harvested. Each brain tissue was divided into two-halves, the right and left hemispheres, from which the hippocampus and cortex were dissected and stored at −80 °C for biochemical analyses. Other samples of the whole brain were stored in 10% formaldehyde for histopathological investigations.

#### 2.8.3 Dose selection

All tested extracts and drugs were dissolved in normal saline. The dose of orientin (10 mg/kg), a flavonoid compound with reported neuroprotective potential, was selected according to the previously reported literature (Tian et al., 2018; Zhong et al., 2019). The quantitative amount of VT (300 mg/kg) and VB (50 mg/kg) were calculated according to the amount of orientin present in the standardized VT and VB extracts, as determined by the UPLC-PDA method. All treatments and vehicles were prepared to be administered at a volume of 10 mL/kg.

#### 2.8.4 Preparation of the brain homogenate

The brain tissue samples (cortex and hippocampus) were subjected to the homogenization process separately. Firstly, the brain tissues were snap-frozen by liquid nitrogen and were crushed till a soft paste was obtained. A PBS of 9x tissue weight was then added to the tissue and mixed well. The mixture was centrifuged at 12 *g* for 15 min, and the supernatant was separated from the tissue residue for further biochemical analyses, including assessment of the AChE inhibitory activity and the concentration of AChE enzyme.

#### 2.8.5 Histopathological examination

The whole brain of the rats was stored in neutral buffered 10% formaldehyde for a subsequent deep investigation of the hippocampal, cortical, and striatal regions. The tissues were embedded in paraffin and cut into sections with 3–4 µm thickness using a microtome. The tissues were further stained using hematoxylin and eosin (H&E). The structure and morphology of the cells were examined using the light microscope at x100, x200, and ×400 magnification powers. The cortex tissues were examined in different terms (meninges, neurons, glial cells, and blood vessels). Meanwhile, striatal neuronal degeneration or background and blood vessels were investigated, in addition to the hippocampal regions, Cornu Ammonis (CA1, CA2, and CA3), dentate gyrus (DG), interneuron area, and blood vessels by the light microscope.

#### 2.8.6 Evaluation parameters

The neuroprotective evaluation parameters encompass two sections.

##### 2.8.6.1 The behavior parameters

###### 2.8.6.1.1 The elevated plus maze test (EPM)

This test was performed according to (Otari et al., 2012; Herrera-Ruiz et al., 2008). The apparatus is in the form of a plus shape (+) composed of two open arms (50 cm × 10 cm) and two enclosed arms (50 cm × 10 cm x 40 cm) with a central platform (10 cm × 10 cm). This test depends on a general preference of rats to remain in enclosed spaces with bound edges rather than open spaces. This phenomenon is called thigmotaxis, which is the antipathy of rodents to open spaces, and this action is applied to the EPM mode as the rats limit their movements by entering one of the closed arms.

On the 8th day of the experiment, the rats first received the scopolamine dose 30 min before the treatment protocol. After another 30 min, the rats were subjected to EPM. The test was carried out by placing each rat in an open arm facing away from the central platform until it entered one of the closed arms.

The time needed for the rat to move into one of the closed arms with all its four limbs was recorded as the latency time (LT) and named (L0). The rat was allowed an acquisition trial of a maximum of 90 s, and then it was allowed to explore the maze for another 20 s (Morales-Delgado et al., 2018). If the rat did not enter a closed arm within 90 s, it was pushed gently toward one of the closed arms, and the LT was recorded herein as 90 s. Animals that failed to stay on the apparatus were excluded. The apparatus was wiped with 70% ethanol after testing each animal. The rats were returned to their home cages, and this learned task was examined after 24 h.

On the 9th day, the task was repeated to examine the retention of the learned task, but each rat was left for only 90 s to enter one of the enclosed arms without time extension, and the time was expressed as L1. The learned task was expressed as an inflexion ratio (IR), which was calculated using the following formula:

$$\text{Inflexion Ratio }\left(\text{IR}\right)=\left(L0-L1\right)/\;L1$$

Where L0 = latency time in the acquisition trial or the time needed for the rats to enter the closed arm within 90 s on the 8th day.

L1 = latency time in the test phase or the time needed for the rats to enter the closed arm after 24 h from the first trial on the 9th day.

Also, the percentage of retention of latency was calculated as L1/L0 x 100.

There is a direct proportional relationship between increasing IR and the improvement of learning and memory status of rats; however, the percentage of retention of latency is inversely proportional to learning and memory skills (Rajesh et al., 2017). Since the rats have antipathy to high and open spaces, the shortened transfer latency (TL), the time taken by each rat to enter the closed arm by its four limbs, on the 2nd day (retention phase) relevant to the TL of the 1st day (acquisition phase) is considered an indicator of enhanced learning skills and memory (Sharma and Kulkarni, 1992).

###### 2.8.6.1.2 The novel object recognition test (NORT)

This test was performed according to (Hoang et al., 2020). The apparatus is composed of black-coated, squared opaque plastic or acrylic material (50 cm × 50 cm × 35 cm) with a grid floor.

**FIGURE 1:**
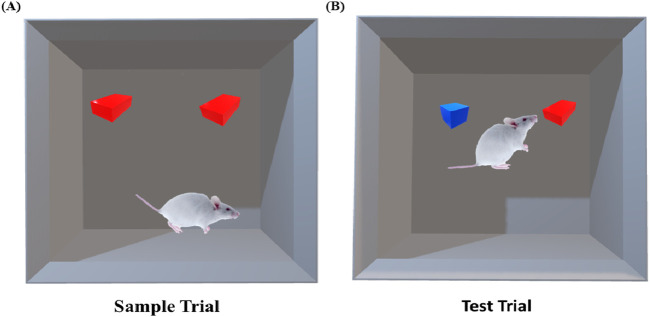
Demonstration of the object recognition test.

On the 8th day, each rat was allowed to explore the box without any object for 3 min for habituation. After each trial, the box was cleaned with 70% ethanol. On the 9th day, the two identical objects with the same color were put in the box opposite each other. In the first (sample) trial, the rats were allowed to explore the two identical objects for a total time of 20 s of exploration of both objects, as illustrated in Figure 1A. The exploration was counted if the rat directed its nose at less than 2 cm. In case of no exploration, the time was extended to 5 min, and the time of total exploration was counted. On the 10th day, one of the two objects was replaced with a new, different object in color and shape. In the second (test) trial, each rat was allowed to explore the objects individually for 3 min, and the time for the exploration of the familiar object (T_f_) and the new object (T_n_) (McGirr et al., 2020) were counted and recorded as illustrated in Figure 1B. The rat that failed to achieve an exploration time of 7 s was excluded.

The discrimination ratio (DR) and the percentage of object interaction were calculated, where the DR = (T_n_-T_f_)/(T_n_ + T_f_) and % of interaction = [T_n_ or T_f_/(T_n_ + T_f_)] x 100. This test assesses long-term memory (after a 24 h retention period) and the ability of the animal to discriminate between novel and old objects and retrieve this information (Moore et al., 2013).

##### 2.8.6.2 Biochemical parameters

###### 2.8.6.2.1 *In vivo* assessment of acetylcholinesterase (AchE) concentration using the ELISA method

Enzyme-linked immunosorbent assay (ELISA) is an extensively adopted universal technique used for the detection and quantification of minute amounts of certain antigens in biological samples (Lequin, 2005; Gan and Patel, 2013). This test was carried out according to the manufacturer’s instructions. It depends on the measurement of the concentration of the AChE enzyme, which is an antigen in the rats’ hippocampus.

The microplate of the ELISA kit was designed to be pre-coated with the specific antibody of rat AChE. Firstly, an aliquot of 1 mL of standard solution and samples (homogenized hippocampus tissues of control, Sco, E150, E300, and VB groups) were diluted using buffer solution to 50 ng/mL. A successive serial dilution of the standard was done to produce the concentrations of (25, 12.5, 6.25, 3.13, 1.57, and 0.78 ng/mL). An aliquot of 100 µL of each concentration of the standard solution was added to the wells in duplicate, and 100 µL of different samples diluted by the sample diluent was added to another well. The plate was sealed and incubated at 37 °C for 90 min. After the incubation period, a biotinylated detection antibody working solution was added to each well, then the plate was covered and incubated at 37 °C for 60 min so that the specific antigen-antibody reaction could be initiated. Any solution in the wells was decanted and washed with a washing buffer. The washing step was repeated three times. An aliquot of horseradish peroxidase (HRP) conjugate working solution was added to each well, and the plate was incubated at 37 °C for 60 min.

After the incubation, the plate was washed as previously mentioned five times. Then, a coloring agent was added to each well, the plate was covered, and incubated at 37 °C for 15 min. Finally, a stop solution was applied to each well to stop the reaction. The optical density (OD) was measured using a microplate reader at 450 nm. Only the wells containing the specific rat AChE antigen that reacted with the biotinylated detection antibody, the HRP conjugate antibody, and the coloring agent produced a color.

The amount of AChE enzyme in the sample is directly proportional to the color intensity. The results were presented and plotted as concentration *versus* optical density.

###### 2.8.6.2.2 *In vivo* assessment of acetylcholinesterase inhibitory activity using Ellman’s microplate assay

Ellman’s microplate is an *in vitro* colorimetric assay that measures the inhibitory activity of the plant extract to acetylcholinesterase (Ellman et al., 1961).

A 96-well microplate was firstly filled with 140 µL of 0.1 M sodium phosphate buffer at pH 8 followed by 20 µL of the homogenized hippocampal and cortical tissues for each animal group (control, Scop, E150, E300, and VB groups) were dissolved in 10% methanol with 2-folded serial dilutions (500, 250, 125, 62.5, 31.25, 15.63, 7.81, and 3.9 μg/mL) then 20 µL of 0.09 unit/mL AChE enzyme was added to the microplate. The microplate was incubated at room temperature for 20 min. After the incubation period, 10 µL of 10 mM DTNB (5,5-dithio-bis-(2-nitrobenzoic acid)) was added to each well, followed by 10 µL of 14 mM acetyl thiocholine iodide (ACTI), which was used as a substrate. The chemical principle of the assay is that the AChE enzyme hydrolyzes ACTI substrate to thiocholine with a yellow color and acetic acid. After the enzymatic reaction, the absorbance of the yellow-colored product was measured at 412 nm for 30 min using a microplate reader (Biotek, USA).

The intensity of the yellow color is directly proportional to enzyme activity. The absorbance of the control that contains the sample dissolved in methanol, DTNB, and ACTI without the addition of AChE enzyme was measured under the same conditions. Donepezil (Sigma Aldrich, St. Louis, US) is an AChE enzyme inhibitor that was used as a positive standard dissolved in methanol and measured at the same concentrations as the sample.

The absorbance of the sample was corrected by subtracting the absorbance of the blank. The inhibitory concentration (IC_50_) of the tested sample and the positive standard was assessed as a 50% reduction of UV absorbance compared *to* the control. The inhibitory activity (nmol/min/mL) was measured as the amount of enzyme that produces 1 nmol of thiocholine per min according to the following equation.

Where B is the amount of TNB (nmol), RT = reaction time, M is the total amount of the sample added into the reaction well (mL), and D is the dilution factor.

#### 2.8.7 Statistical analysis

Statistical analysis for behavioral tests and AChE concentration was calculated by GraphPad Prism (v9) using one-way analysis of variance (ANOVA) followed by Tukey as a post-hoc test and tests available in GraphPad Prism (version 9), including the Brown–Forsythe test. Values were presented as a mean with 95% confidence interval (CI), and a significant level was considered at a p-value <0.05. For UPLC-PDA analysis, regression equations and regression coefficient (R^2^) were calculated and illustrated in Table 3.

**TABLE 3:** Standard calibration curves and validation parameters of isoorientin, orientin-2´´-*O-α*-L-rhamnoside, and orientin using the proposed ultra-performance liquid chromatography (UPLC) method.

Peaks	Regression equation*	Slope	Intercept	R^2^	LOD	LOQ	Recovery (mean ± SD)
Isoorientin	*y = 1.243x+1.9433*	0.1956	+0.7861	0.999	4.52	13.685	100.608 ± 1.12
Orientin-2´´-*O-α*-L-rhamnoside	*y = 0.7482x - 0.3007*	0.7482	−0.3007	0.996	4.2	12.716	99.7 ± 0.237
Orientin	*y = 0.1291x - 0.0036*	0.1291	−0.0036	0.998	4.326	13.086	99.64 + 0.65

* The regression equation was calculated from the average peak area.

## 3 Results and discussion

### 3.1 Identification of isolated compounds

Six pure compounds (**V1**-**V6**) were isolated from *V. pubescens* bark from two active fractions, *viz.*, DCM and *n*-BuOH fractions. The structures were elucidated using 1D NMR spectroscopy (^1^H and ^13^C NMR) as presented in Supplementary Figure S1-11, supplementary material.

Upon comparing the isolated compounds with those in literature values, the pentacyclic triterpene betulinic acid (**V1**) (Tijjani et al., 2012) and the phenolic acids vanillic acid (**V2**) (Zhang et al., 2009) and *P*-hydroxybenzoic acid (**V3**) (Li et al., 2013; Scott, 1972) were isolated from the DCM fraction. Furthermore, three flavonoids were isolated from the *n*-BuOH fraction, including orientin-2´ˊ-*O*-*α*-L-rhamnoside (luteolin-8-*C*-*β-*D-glucoside-2´ˊ-*O*-*α*-L-rhamnoside) (**V4**) (Prinz et al., 2007), isoorientin (luteolin-6-*C*-glucoside) (**V5**) (Harborne and Mabry, 2013; Agrawal, 2013; Lu et al., 2019), and orientin (luteolin-8-*C*-glucoside) (**V6**) (Harborne and Mabry, 2013; Agrawal, 2013; Lu et al., 2019).

**FIGURE 2:**
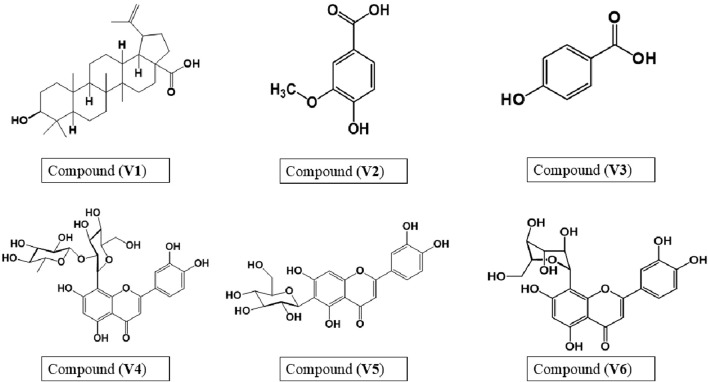
Chemical structures of isolated compounds from *V*. *pubescens* bark. **V1**: betulinic acid; **V2**: vanillic acid; **V3**: *p*-hydroxybenzoic acid; **V4**: orientin-2´ˊ-*O*-*α*-L-rhamnoside**; V5:** isoorientin; **V6:** orientin.

The chemical structures of isolated compounds are depicted in Figure 2. This study presented the first attempt to isolate vanillic acid, isoorientin, orientin-2´ˊ-*O*-*α*-L-rhamnoside, and orientin for the first time from *V. pubescens* bark besides the previously isolated compounds, *viz*., betulinic acid and *p*-hydroxybenzoic acid (Anwar, 2019).

### 3.2 Ultra-performance liquid chromatography-photodiode array (UPLC-PDA)

**FIGURE 3:**
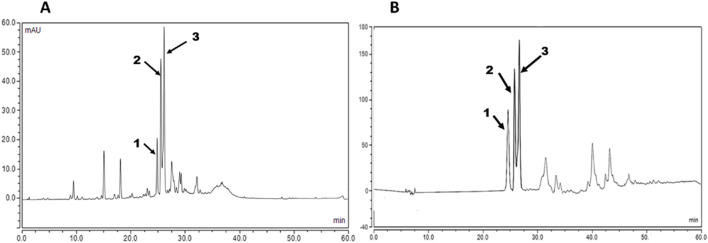
Ultra-performance liquid chromatography-photodiode array (UPLC-PDA) chromatograms of **(A)** total methanol extract, **(B)** *n*- BuOH of *V. pubescens* bark, *λ* = 340 nm. Peaks **(1)** isoorientin, **(2)** orientin-2´´-*O-α*-L-rhamnoside, and **(3)** orientin.

**FIGURE 4:**
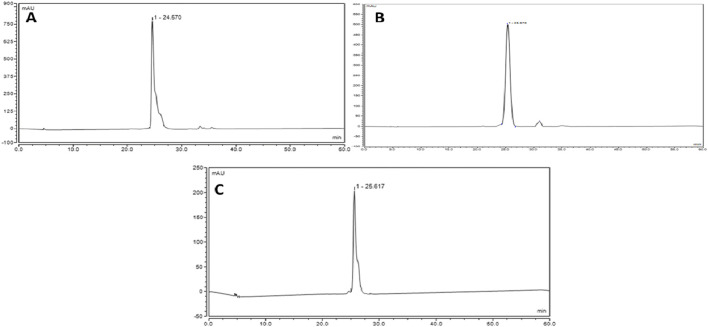
Ultra-performance liquid chromatography-photodiode array (UPLC-PDA) chromatograms of the isolated flavonoid C-glycosides **(A)** isoorientin, **(B)** orientin-2´´-*O-α*-L-rhamnoside, and **(C)** orientin representing the retention time (RT) of each peak.

Qualitative analysis is implemented to obtain fingerprints of the total extract and fractions (Sun et al., 2017). Under these optimized conditions, the adopted method delivered chromatographic separations of standard mixtures and the chemical profiles of the total methanol extract and *n*-BuOH fraction were qualitatively assigned in chromatograms Figures 3, 4.

The chromatograms of *n*-BuOH showed accumulations of the high-intensity flavonoid-C-glycoside peaks. The major peaks were eluted and identified *via* comparison of the retention time (RT) with the retention time of the isolated flavonoids. The results showed that the compounds appeared at the RT of isoorientin (Peak **1**, 24.570 min), orientin-2´´-*O-α*-L-rhamnoside (Peak **2**, 25.373 min), and orientin (Peak **3**, 25.617 min). All examined standards were found in the total methanol extract and *n*-BuOH fraction. The results were confirmed using the UV spectrum profiles of the studied analytes using the PDA of the UPLC.

**FIGURE 5:**
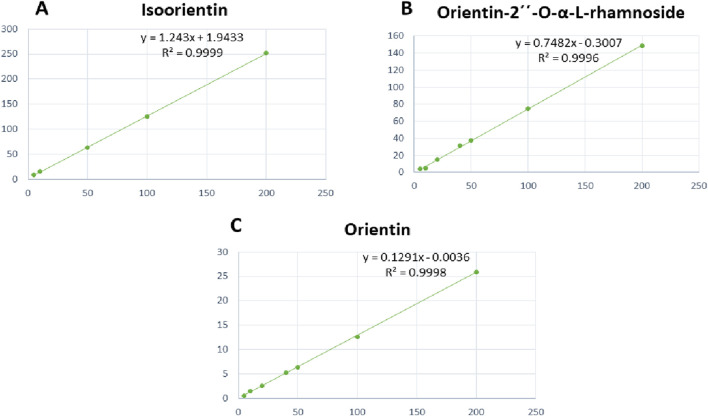
Standard calibration curves of **(A)** isoorientin, **(B)** orientin-2´´-*O-α*-L-rhamnoside, and **(C)** orientin.

For the quantitative analysis, linearity was accomplished by preparing a calibration set for isoorientin, orientin-2´´-*O-α*-L-rhamnoside, and orientin concentration ranging from 50–200 μg/mL. Each concentration was injected into the UPLC under the optimum conditions in triplicate, and then the calibration curve was plotted for each peak using the peak area against the corresponding concentration. Calibration curves for isoorientin, orientin-2´´-*O-α*-L-rhamnoside, and orientin, as shown in Figure 5, showed that the method was linear in the proposed range for the three compounds.

According to the calculated regression equations, the quantification of the major flavonoid *C*-glycosides was constructed. The results showed a high accumulation of orientin was observed in the total extract and *n*-BuOH fractions, which represented 7.85% and 26.8%, respectively. The second major compound was orientin-2´´-*O*-*α*-L-rhamnoside, representing 1.8% of the total extract and 9.2% of the *n*-BuOH fraction. Meanwhile, isoorientin represented 1.2% and 2.3% of the total extract and *n*-BuOH fractions, respectively. The validation parameters of the UPLC method, including linearity (R^2^), limit of detection (LOD), limit of quantification (LOQ), and recovery (mean ± SD), are summarized in Table 3.

Flavonoids are promising natural candidates for acetylcholinesterase (AChE) inhibition, largely due to the presence of free hydroxyl groups that contribute to their notable inhibitory activity (Khan et al., 2018). Furthermore, the quantitative analysis using UPLC-PDA showed the richness of *V. pubescens* defatted methanol extract and *n*-buOH fraction with the isolated flavonoid, orientin, which has been reported to have anti-inflammatory, antioxidant, and neuroprotective activities (Zhong et al., 2019). Thus, this study examined the anti-dementia potential of the total defatted methanol extract and the *n*-BuOH fraction of *V. pubescens* bark alongside the isolated orientin compound using scopolamine-ameliorated dementia in male Sprague-Dawley rats as an *in vivo* model.

### 3.3 Biological study

#### 3.3.1 Histopathological examination

**FIGURE 6:**
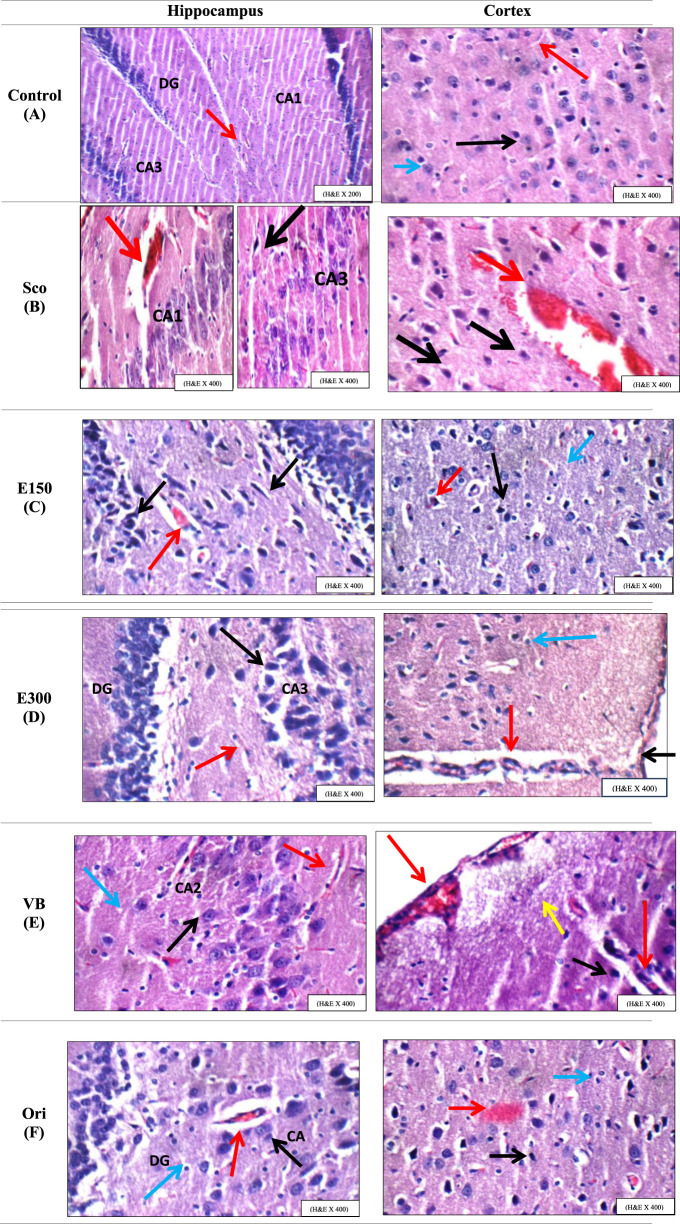
**(A)** showing normal brain section; **(B)** group injected with Sco without treatment; **(C)** rats injected with Sco and treated with 150 mg/kg of total extract E150; **(D)** rats injected with Sco and treated with 300 mg/kg of total extract E300; **(E)** rats injected with Sco and treated with *n*-BuOH fraction; **(F)** rats injected with Sco and treated with orientin. Black arrow: pyramidal neurons, blue arrow: inter-neuron area, CA: Cornu Amonis, DG: dentate gyrus, Ori: orientin, red arrow: blood vessels, Sco: scopolamine, VB: BuOH fraction of Vitex pubescens bark, and yellow arrow: mild sub-meningeal edema.

Scopolamine was used to induce dementia in rats as it can impair cognitive functions, destroy the cholinergic centers, and have detrimental histopathological changes in the hippocampus and cortex tissues (An et al., 2019). All experimental groups first received scopolamine for dementia induction and then received the required treatment regimen. Histopathological examination was carried out to record the detrimental effects of scopolamine in the hippocampus, cortex, and striatum tissues, as well as to evaluate the effect of the treatments to counteract scopolamine effects and restore the normal histological features of the brain compared to scopolamine and control rat groups, as illustrated in Figure 6 and summarized in Tables 4 & 5.

**TABLE 4:** Summary of the histopathological changes displayed in the hippocampus tissue.

​	Hippocampus					
	CA1	CA2	CA3	DG	Inter-neuron area	Blood vessels
Gp1: Control	0	0	0	0	0	0
Gp2: SCO	0	+	+	+	0	++ in CA1, + in DG
Gp3: E 150	0 to +	0 to +	0 to +	0 to +	0	0
Gp4: E 300	0 to +	0	0	0 to +	0 to + in DG	0 to + in DG
Gp5: VB	0	0	0	0	0	0 to + in CA3 and DG
Gp6: Ori	0	0	0	0	0	0

0: Average, +: scattered degenerated neurons in Cornu Ammonis (CA) regions; intraneuronal eosinophilic plaque-like areas; mildly dilated or congested blood vessels, ++: Markedly degenerated neurons in CA, regions; markedly dilated blood vessels.

**TABLE 5:** Summary of the histopathological changes displayed in the cortex and striatum.

​	Cortex				Striatum		
	Meninges	Neurons	Blood vessels	Background	Neurons	Blood vessels	Background
Gp1: Control	0	0	0	0	0	0	0
Gp2: SCO	0	+ to ++	+ to ++	0	++	0	0
Gp3: E 150	+	0 to +	0 to +	0	+	0	0
Gp4: E 300	0	+	0	+	+	0	+
Gp5: VB	0	0 to +	0	0	0	0	0
Gp6: Ori	0	0 to +	0 to +	+	+	+	0

0: Average, +: Detached meninges; scattered degenerated neurons; mildly dilated or congested blood vessels; or eosinophilic plaque-like areas in the background, ++: Markedly degenerated neurons; Markedly dilated or congested blood vessels.

The control group (GP-I) showed normal architecture of brain tissues displaying normal neurons (black arrow) with average blood vessels (red arrow), interneural area, glial cells, and meningeal cells (blue arrow) in the hippocampus, cortex, and striatum (Figure 6A).

The scopolamine group (GP-II) showed scattered degenerated pyramidal neurons and mildly to markedly congested blood vessels (red arrow) in DG and CA1, respectively, in the hippocampus tissues. Moreover, the cerebral cortex revealed mildly to markedly degenerated neurons (black arrow) and congested intracerebral blood vessels (red arrow), while the striatal tissue showed markedly degenerated neurons (Figure 6B).

Regarding the treatment groups (E150 and E300), the hippocampus tissues showed a protective effect of the extract against the deficits induced by scopolamine in the two dose levels, as evidenced by average blood vessels (red arrow) and normal pyramidal neurons (black arrow) in all hippocampal regions, with some scattered neurodegeneration observed in a few sections (Figures 6C,D). Upon examination of cortical tissues, the E150 group showed average glial cells (blue arrow), normal to mildly congested blood vessels (red arrow), with average scattered degenerated neurons, and detached meninges (black arrow). In the cortex of the E300-treated group, the meninges (black arrow) and blood vessels (red arrow) appeared to have normal architecture with some eosinophilic plaque-like areas and scattered degenerated neurons (blue arrow). Meanwhile, the striatum revealed some scattered neurodegeneration in E150 and E300, with some eosinophilic plaque-like areas in the E300 group.

A noticeable improvement in hippocampal tissues was observed in groups treated with *n*-BuOH fraction and orientin (VB and Ori). Concerning the *n*-BuOH fraction-treated group (VB), normal pyramidal neurons (black arrow), inter-neuron area (blue arrow), and blood vessels (red arrow), with normal to mildly congested blood vessels in DG and CA3 regions only, were observed. The orientin group exhibited obvious protection from scopolamine-induced changes in all areas of the hippocampal tissues. Notably, the *n*-BuOH fraction-treated group showed an advanced effect in the brain cortical and striatal tissues rather than the orientin group. This enhancement included average meninges, average glial cells, blood vessels (red arrow), and a neuronal background area with normal to scattered degenerated cortical neurons (black arrow). The orientin group, however, showed average glial cells, average meninges of the cortex, and a normal background in the striatum, accompanied by normal scattered degenerated neurons (black arrow) with eosinophilic plaque-like areas in the cortex (red arrow) and mildly congested blood vessels in the cortex and striatum.

The stained hippocampus tissue of each examined group (control, Sco, E150, E300, VB, and Ori) was evaluated in terms of average Cornu Amonis (CA1), (CA2), (CA3), normal dentate gyrus (DG), pyramidal neurons, blood vessels, and interneural area. The cortical tissue was evaluated through the cortex and striatum tissues in terms of average meninges neurons, glial cells, and blood vessels.

### 3.4 Evaluation parameters

#### 3.4.1 The behavior parameters

##### 3.4.1.1 The elevated plus maze test (EPM)

**FIGURE 7:**
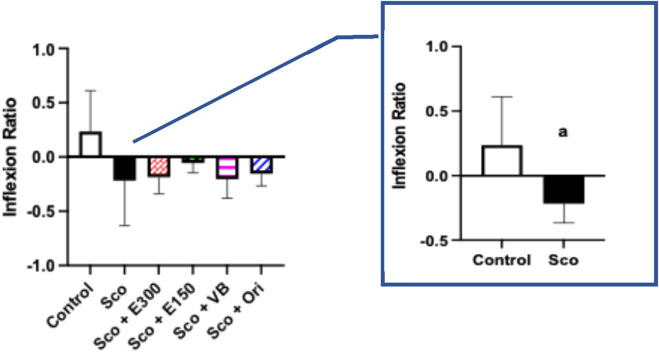
The effect of different treatments on learning skills and memory was investigated in the elevated plus maze (EPM) test The data represent the mean with a 95% confidence interval (CI) of the effect of the treatments of concern on the learning skills and memory demonstrated by the inflexion ratio. The statistical analysis was performed using one-way analysis of variance (ANOVA), followed by Tukey *post-hoc* test (n = 6-7 rat/group). ^a^ sig. diff. *vs* the control group was assessed by a one-tailed Student’s t-test, *p* < 0.05 (as shown in the right plot). Sco: scopolamine; E300: gp. treated with the methanol extract (300 mg/kg/day, p.o.) after scopolamine; E150: gp. treated with the extract (150 mg/kg/day, p.o.) after scopolamine; VB: gp. treated with *n*-BuOH fraction (50 mg/kg/day, p.o.) after scopolamine; Ori: gp. treated with orientin (10 mg/kg/day, p.o.) after scopolamine.

In the current study, the effect of extracts on the learning skills and memory in rats was assessed using the elevated plus maze (EPM) as presented in Figure 7; the TL was recorded first on the 8th day and after a retention time of 24 h, and the inflexion ratio (IR) was calculated. Data showed a significant reduction in rats subjected to scopolamine without any treatments compared to the control group (*p = 0.0298*) using a one-tailed t-test. This implied that scopolamine was capable of inducing memory deficit in rats compared to normal animals.

Upon treatment with E150, E300, VB, and Ori, it was noted that all treatments had the same effect as they showed no significant difference among each other with the highest IR in the E150-treated group; however, this change did not reach a significant level *vs*. the untreated group (sco gp.).

These data may indicate that the most promising treatment among our studied ones that can enhance learning skills and memory is the methanol extract in its smaller dose (150 mg/kg). Though its effect was not statistically significant, there was a tendency toward an increase in the IR after treatment with E150.

Notably, this mild enhancement in cognition and memory might be independent of the dose, as the E300 unexpectedly exhibited a lower IR than E150, noting that this did not reach statistically significant levels. It is believed that the small sample size affected the results negatively, as reaching a meaningful difference between groups was not obvious, though there was a trend of difference among the treated groups.

##### 3.4.1.2 The novel object recognition test (NORT)

It was found that rats injected with scopolamine had a significantly lower DR than the control group (*p* = 0.0068). This indicated an impairment of memory in rats induced by scopolamine.

**FIGURE 8:**
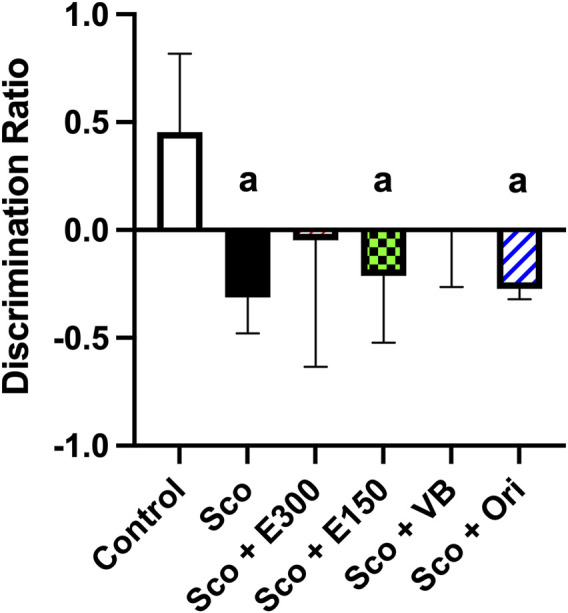
The effect of different treatments on long-term memory investigated in the novel object recognition test (NORT) The data represent the mean with a 95% confidence interval (CI) of the effect of the treatments of concern on long-term memory, as demonstrated by the discrimination ratio. The statistical analysis was performed using one-way analysis of variance (ANOVA), followed by the Tukey *post-hoc* test. ^a^ sig. diff. at p-value <0.05. Sco: scopolamine; E300: gp. treated with the methanol extract (300 mg/kg/day, p.o.) after scopolamine; E150: gp. treated with the extract (150 mg/kg/day, p.o.) after scopolamine; VB: gp. treated with *n*-BuOH fraction (50 mg/kg/day, p.o.) after scopolamine; Ori: gp. treated with orientin (10 mg/kg/day, p.o.) after scopolamine.

Meanwhile, the tested groups showed the same discrimination index among themselves but with different degrees since the groups injected with *n*-BuOH fraction and E300 had the highest DR compared to the other two treatments, E150 and orientin, to the extent that their means were not significantly different from the control group. On the contrary, E150 and Ori groups showed the lowest DR, which was significantly lower than the mean of the control group (*p* = 0.0238 and 0.0112, respectively). One may deduce that the methanol extract at the dose of 300 mg/kg and *n*-BuOH fraction could improve recognition and long-term memory better than the extract in its small dose (150 mg/kg) and orientin as E300 and *n*-BuOH fraction might approach the normal levels, as shown in Figure 8.

These results did not negate the probability that these extracts may reveal obvious effects if the sample size were bigger and if they were tested in short-term or intermediate memory with shorter retention time. It was reported that NORT has some limitations related to the strains of the animals used, since some animals are noticed to be resistant to the NORT due to visual inability caused by a genetic mutation. With these strains of animals, it would be preferred to use a short retention time rather than 24 h. Moreover, it might be more appropriate to test the preference of the animals toward the object’s shape and size before running the experiment, to confirm their curiosity to explore them, and to test their discrimination ability roughly (Lueptow, 2017). All these factors might explain the outcome here, which barely manifested the difference between the treatments among the groups and in comparison to the scopolamine group.

#### 3.4.2 Biochemical parameters

##### 3.4.2.1 1*. In vivo* assessment of hippocampal acetylcholinesterase (AChE) concentration using ELISA

**FIGURE 9:**
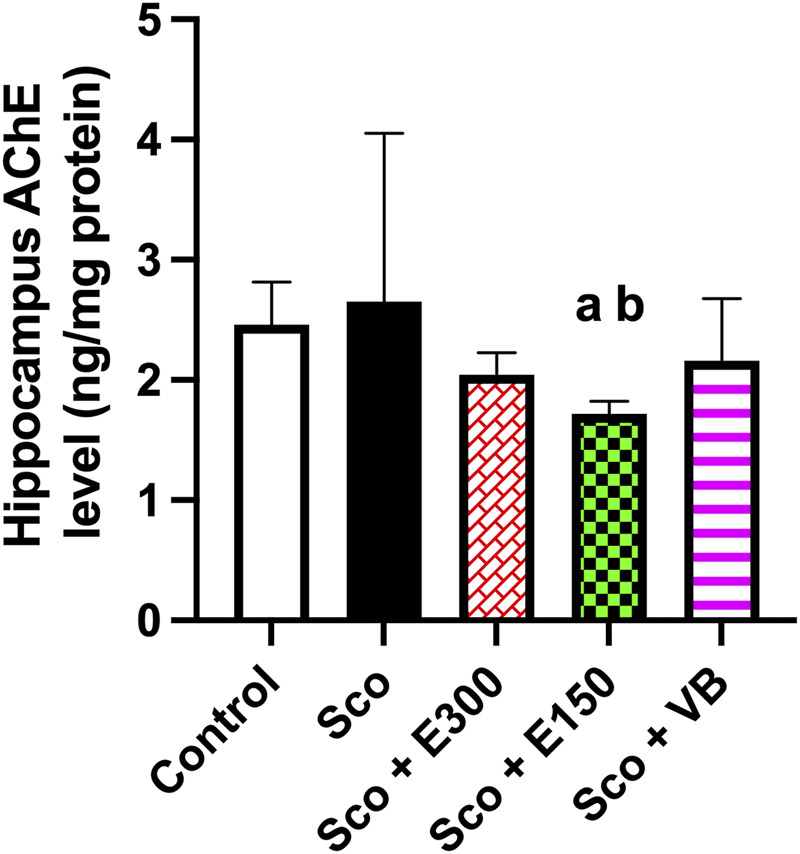
The effect of the studied treatments on acetylcholinesterase (AChE) level per mg protein in hippocampus tissue (n = 5) The plot represents the mean with a 95% confidence interval (CI) of the effect of the treatments of concern on the concentration of hippocampal AChE. The statistical analysis was performed using one-way analysis of variance (ANOVA), followed by Tukey’s *post-hoc* test. ^a^ sig. diff. *vs*. control group; ^b^ sig. diff. *vs*. scopolamine group at p-value <0.05. Sco: scopolamine untreated group; E300: gp. treated with 300 mg/kg/day of total extract after scopolamine; E150: gp. treated with 150 mg/kg/day of total extract after scopolamine; VB: gp. treated with *n*-BuOH fraction after scopolamine.

The assessment of AChE activity and concentration was carried out as depicted in Figure 9. The scopolamine group showed the highest concentration of AChE among all treated groups, which indicated the ability of scopolamine to induce dementia (Rahiman et al., 2015).

Regarding the treatment groups, it was observed that the E150 group induced a significant decrease in AChE concentration compared to scopolamine (*p* = 0.0153) as well as the control group (*p* = 0.0481). Meanwhile, the E300 and *n*-BuOH fraction-treated groups exhibited the same effect in lowering the AChE level in the hippocampus, which did not differ significantly from scopolamine and control groups. This could highlight the efficiency of the total extract of *V. pubescens* to suppress AChE levels in hippocampus tissue, whereas this detrimental effect was not in a dose-dependent manner.

##### 3.4.2.2 *In vivo* assessment of acetylcholinesterase activity using Ellman’s microplate assay

AChE inhibitory activity has been assessed *in vivo* using Ellman’s assay. It was concluded that the scopolamine group induced the highest activity of AChE enzyme, which reflected that the model was accomplished, and scopolamine induced the neuronal deficit compared to the control group.

**FIGURE 10:**
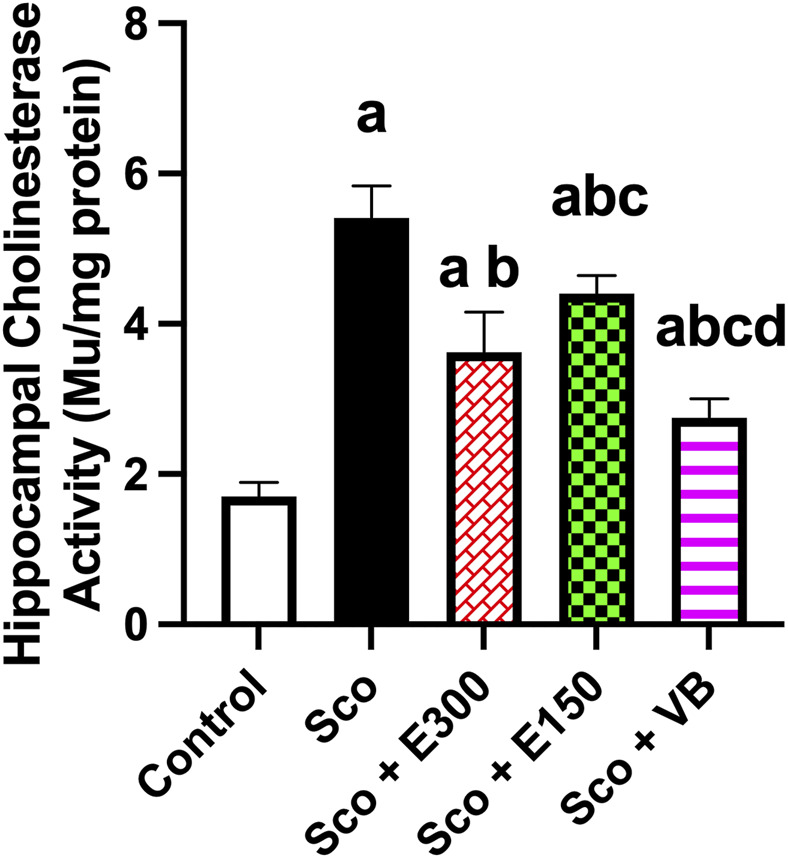
The effect of the treatments studied on acetylcholinesterase (AChE) activity in hippocampus tissue (n = 4). The plot represents mean with 95% CI of the effect of treatments of concern on the concentration of AChE. The statistical analysis was performed using one-way ANOVA, followed by Tukey’s *post-hoc* test. The significance level was at *p*-value <0.05. a sig. diff. vs. control group; b sig. diff. vs. scopolamine group, c sig. diff. vs. E300, d sig. diff. vs. E150. Sco: scopolamine untreated group; E300: gp. treated with 300 mg/kg of total extract after scopolamine; E150: gp. treated with 150 mg/kg of total extract after scopolamine; But: gp. treated with n-BuOH fraction after scopolamine.

The *n*-BuOH fraction-treated group exhibited the most significant inhibition of AChE among all treatment groups compared to the scopolamine group. Moreover, the E300 group exhibited significant activity compared to E150. Thus, it was concluded that all treatment groups had anticholinesterase inhibitory activity, and they ameliorated the dementia induced by scopolamine as illustrated in Figure 10. Furthermore, the effect of the methanol extract appeared to be dose dependent.

Considering the prior outcomes, the methanol extract of *V. pubescens* showed promising anticholinesterase activity *in vivo*, in a dose-dependent manner. Moreover, the *n*-BuOH fraction displayed inhibitory activity against the AChE enzyme, superior to that of the total extract.

These effects were also evident histopathologically, demonstrating potential to ameliorate the deficits induced by scopolamine in the hippocampus, cortex, and striatum. E150 showed the highest ability to reduce the level of AChE in the hippocampal tissue of the rats in the scopolamine-induced model of dementia. The total extract exhibited a dose-dependent effect regarding enhancement of long-term or recognition memory in rats in NORT, while a dose-independent effect was seen in the learning and memory of rats in EPM, though these effects did not reach a statistically significant level. Therefore, further behavioral tests need to be done on a large sample size to confirm the effects of the studied extracts on behavior.

In summary, the phytochemical investigation of bioactive fractions derived from *V. pubescens* bark led to the isolation of six compounds, four of which were isolated from *V. pubescens* bark for the first time, *viz*. phenolic acid (vanillic acid, **V2**) and flavonoid-*C*-glycosides (**V4-6**) isoorientin, orientin-2´´-*O-α*-L-rhamnoside, and orientin alongside the previously isolated betulinic acid and *p-*hydroxybenzoic acid. The UPLC-PDA was utilized for qualitative and quantitative analyses, revealing the richness of *V. pubescens* extract with flavonoid-*C*-glycosides, especially orientin (luteolin-8-*C*-glycoside), constituting about 7.85% and 26.8% of the total defatted methanol extract and *n*-BuOH fraction, respectively, followed by orientin-2´´-*O-α*-L-rhamnoside and then isoorientin.

Regarding the biological activity, this work represents the first attempt to investigate the anti-dementia potential of the defatted methanol extract, *n*-BuOH fraction of *V. pubescens* bark, and orientin in scopolamine-induced dementia in male Sprague-Dawley rat model. Notably, both the total defatted methanol extract and *n*-BuOH fraction exhibited promising anti-dementia activity. It is worth mentioning that the *n*-BuOH fraction exhibited superior inhibitory activity of AchE compared to the total methanol extract in two doses. Furthermore, histopathological examination of the cortical and hippocampal tissues confirmed that the *n*-BuOH fraction exhibited the advanced ability to mitigate the neuronal deficits induced by scopolamine.

## 4 Conclusion

In conclusion, the results highlight the therapeutic potential of *V. pubescens* defatted methanol extract, particularly the *n*-BuOH fraction, in combating neurodegenerative disorders such as Alzheimer’s disease. These findings suggest that the *n*-BuOH fraction and orientin merit further investigation as promising candidates for the development of natural anti-dementia therapeutics. Despite these promising results, several limitations should be acknowledged. First, the neuroprotective activity was evaluated using a scopolamine-induced dementia model. This model mainly reflects cholinergic dysfunction and does not fully capture the complex causes of Alzheimer’s disease, such as amyloid-*β* buildup and *tau* pathology. Additionally, the behavioral findings did not consistently show statistical significance due to the relatively small sample size. Moreover, the long-term toxicity, pharmacokinetic studies, and clinical use of the defatted methanol extract and *n*-BuOH fraction were not assessed in this study. Future investigation should focus on elucidating the complex molecular mechanisms underlying the neuroprotective effects of *Vitex pubescens* bark extracts. This includes evaluating biomarkers of oxidative stress, inflammatory responses, amyloid-*β,* and apoptosis, as well as assessing gene and protein expression related to cholinergic function and neurodegeneration. This will provide evidence on their potential to treat Alzheimer’s disease. The successful development of *Vitex pubescens* bark extracts as anti-dementia therapeutics will ultimately depend on well-designed preclinical and clinical studies.

## Data Availability

The original contributions presented in the study are included in the article/Supplementary Material, further inquiries can be directed to the corresponding author.

## Footnotes

1 (http://ethnoherbs.net/).

## Glossary

AD: Alzheimer’s disease
AchE: Acetylcholinesterase
DALYs: Disability-adjusted life-years
Ach: Acetylcholine
Sco: Scopolamine
FDA: Food and Drug Administration
*n*-BuOH: *n*-Butanol
EtOAc: Ethyl acetate
DCM: Dichloromethane
DMSO: Deuterated dimethyl sulphoxide
**CD**_**3**_ **OD**: Deuterated methanol
VLC: Vacuum liquid chromatography
TLC: Thin layer chromatography
**CO**_**2**_: Carbon dioxide
H&E: Hematoxylin and eosin
CA: Cornu Ammonis
DG: Dentate gyrus
EPM: Elevated plus maze test
LT: Latency time
IR: Inflexion ratio
NORT: Novel object recognition test
ELISA: Enzyme-linked immunosorbent assay
HRP: Horseradish peroxidase
OD: Optical density
ACTI: Acetyl thiocholine iodide
DTNB: 5,5-Dithio-bis-(2-nitrobenzoic acid)
IC50: Half maximal inhibition concentration
RT: Retention time
UPLC: PDA Ultra-Performance Liquid Chromatography Photodiode Array
TL: Transfer latency
DR: Discrimination ratio

## References

[B1] Abdelbaset S. El-Kersh D. M. Ayoub I. M. Eldahshan O. A. (2023). GC-MS profiling of *Vitex pinnata* bark lipophilic extract and screening of its anti-TB and cytotoxic activities. Nat. Prod. Res. 37, 2718–2724. 10.1080/14786419.2022.2124512 36110061

[B2] Abdelbaset S. Ayoub I. M. Mohamed O. G. Tripathi A. Eldahshan O. A. El-Kersh D. M. (2024). Metabolic profiling of *Vitex Pubescens* Vahl bark *via* UPLC-ESI-QTOF/MS/MS analysis and evaluation of its antioxidant and acetylcholinesterase inhibitory activities. BMC Complementary Medicine Therapies 24, 232. 10.1186/s12906-024-04520-3 38877470 PMC11177471

[B3] Abdelwahed M. T. Hegazy M. A. Mohamed E. H. (2023). A validated green UPLC method for the quantification of synthetic pharmaceutical adulterants in the extract of the aphrodisiac herbal plant ashwagandha (*Withania somnifera*) and in spiked human plasma. Sustain. Chem. and Pharm. 36, 101297. 10.1016/j.scp.2023.101297

[B4] Agrawal P. K. (2013). Carbon-13 NMR of flavonoids. Elsevier.

[B5] Al-Akwaa A. A. Asmawi M. Z. Dewa A. Mahmud R. (2020). Antihypertensive activity and vascular reactivity mechanisms of *Vitex pubescens* leaf extracts in spontaneously hypertensive rats. Heliyon 6, e04588. 10.1016/j.heliyon.2020.e04588 32775735 PMC7399130

[B6] An K. S. Choi Y. O. Lee S. M. Ryu H. Y. Kang S. J. Yeon Y. (2019). Ginsenosides Rg5 and Rk1 enriched cultured wild ginseng root extract bioconversion of *Pediococcus pentosaceus* HLJG0702: effect on scopolamine-induced memory dysfunction in mice. Nutrients 11, 1120. 10.3390/nu11051120 31137483 PMC6566503

[B7] Anwar L. (2019). Structure elucidation of a pentacyclic triterpenoid and phenolic from steam bark of *Vitex Pubescens* vahl. J. Chem. Nat. Resour. 1, 68–74. 10.32734/jcnar.v1i1.837

[B8] Anwar L. Ibrahim S. Putra D. Efdi M. (2015). LABDANE-type diterpenoid and phenolic from the stem bark of Vitex pubescens Vahl. J. Chem. Pharm. Res. 7, 290–294.

[B9] Anwar L. Santoni A. P.Putra D. Efdi M. (2019). Cytotoxic lactone-type diterpenoids and triterpenoid from *Vitex pubescens* Vahl. Rasāyan J. Chem. 12, 1641–1645. 10.31788/RJC.2019.1235025

[B10] Brusotti G. Cesari I. Dentamaro A. Caccialanza G. Massolini G. (2014). Isolation and characterization of bioactive compounds from plant resources: the role of analysis in the ethnopharmacological approach. J. Pharmaceutical and Biomedical Analysis 87, 218–228. 10.1016/j.jpba.2013.03.007 23591140

[B11] El-Ganainy S. O. Gowayed M. A. Agami M. Mohamed P. Belal M. Farid R. M. (2021). Galantamine nanoparticles outperform oral galantamine in an Alzheimer’s rat model: pharmacokinetics and pharmacodynamics. Nanomedicine 16, 1281–1296. 10.2217/nnm-2021-0051 34013783

[B12] Ellman G. L. Courtney K. D. Andres V. Feather-Stone R. M. (1961). A new and rapid colorimetric determination of acetylcholinesterase activity. Biochem. Pharmacology 7, 88–95. 10.1016/0006-2952(61)90145-9 13726518

[B13] Faisal H. U. Efdi M. Itam A. Okselni T. Anwar L. (2022). Antioxidant activity-guided isolation of phenolic compounds from leaves of *Vitex pinnata* (Lamiaceae). Chem. Sci. Int. J. 31, 1–11. 10.9734/CSJI/2022/v31i6824

[B14] Gamal El-Din M. I. Youssef F. S. Ashour M. L. Eldahshan O. A. Singab A. N. B. (2018). Comparative analysis of volatile constituents of *Pachira aquatica* Aubl. and *Pachira glabra* Pasq., their anti-Mycobacterial and anti-helicobacter pylori activities and their metabolic discrimination using chemometrics. J. Essent. Oil Bear. Plants 21, 1550–1567. 10.1080/0972060X.2019.1571950

[B15] Gan S. D. Patel K. R. (2013). Enzyme immunoassay and enzyme-linked immunosorbent assay. Invest. Dermatol 133, e12. 10.1038/jid.2013.287 23949770

[B16] Gustavsson A. Norton N. Fast T. Frölich L. Georges J. Holzapfel D. (2023). Global estimates on the number of persons across the Alzheimer's disease continuum. Alzheimer's and Dementia 19, 658–670. 10.1002/alz.12694 35652476

[B17] Hampel H. Mesulam M. M. Cuello A. C. Farlow M. R. Giacobini E. Grossberg G. T. (2018). The cholinergic system in the pathophysiology and treatment of Alzheimer’s disease. Brain 141, 1917–1933. 10.1093/brain/awy132 29850777 PMC6022632

[B18] Hansen R. A. Gartlehner G. Webb A. P. Morgan L. C. Moore C. G. Jonas D. E. (2008). Efficacy and safety of donepezil, galantamine, and rivastigmine for the treatment of Alzheimer’s disease: a systematic review and meta-analysis. Clin. Interventions Aging 3, 211–225. 10.2147/cia.S12159936 18686744 PMC2546466

[B19] Harborne J. B. Mabry T. J. (2013). The flavonoids: advances in research.

[B20] Herrera-Ruiz M. Román-Ramos R. Zamilpa A. Tortoriello J. Jiménez-Ferrer J. E. (2008). Flavonoids from Tilia americana with anxiolytic activity in plus-maze test. J. Ethnopharmacol. 118, 312–317. 10.1016/j.jep.2008.04.019 18539420

[B21] Hoang T. H. X. Ho D. V. Van Phan K. Le Q. V. Raal A. Nguyen H. T. (2020). Effects of *Hippeastrum reticulatum* on memory, spatial learning and object recognition in a scopolamine-induced animal model of Alzheimer’s disease. Pharm. Biology 58, 1107–1113. 10.1080/13880209.2020.1841810 33170051 PMC7671694

[B22] Hosseinkhani A. Sahragard A. Namdari A. Zarshenas M. M. (2017). Botanical sources for Alzheimer’s: a review on reports from traditional Persian medicine. Am. J. Alzheimer's Dis. Other Dementias 32, 429–437. 10.1177/1533317517717013 28683559 PMC10852953

[B23] Khan H. Marya Amin S. Kamal M. A. Patel S. (2018). Flavonoids as acetylcholinesterase inhibitors: current therapeutic standing and future prospects. Biomed. Pharmacother. 101, 860–870. 10.1016/j.biopha.2018.03.007 29635895

[B24] Kim J. Seo Y. H. Kim J. Goo N. Jeong Y. Bae H. J. (2020). Casticin ameliorates scopolamine-induced cognitive dysfunction in mice. J. Ethnopharmacol. 259, 112843. 10.1016/j.jep.2020.112843 32380246

[B25] Knopman D. S. Amieva H. Petersen R. C. Chételat G. Holtzman D. M. Hyman B. T. (2021). Alzheimer disease. Nat. Reviews Dis. Primers 7, 33. 10.1038/s41572-021-00269-y 33986301 PMC8574196

[B26] Lequin R. M. (2005). Enzyme immunoassay (EIA)/enzyme-linked immunosorbent assay (ELISA). Clin. Chemistry 51, 2415–2418. 10.1373/clinchem.2005.051532 16179424

[B27] Li S. Qiu S. Yao P. Sun H. Fong H. H. S. Zhang H. (2013). Compounds from the fruits of the popular European medicinal plant *Vitex agnus-castus* in chemoprevention *via* NADP (H): quinone oxidoreductase type 1 induction. Evidence-Based Complement. Altern. Med. 2013, 432829. 10.1155/2013/432829 23662135 PMC3638617

[B28] Li Q. He S. Chen Y. Feng F. Qu W. Sun H. (2018). Donepezil-based multi-functional cholinesterase inhibitors for treatment of Alzheimer's disease. Eur. Journal Medicinal Chemistry 158, 463–477. 10.1016/j.ejmech.2018.09.031 30243151

[B29] Lu Y. Zhu S. He Y. Peng C. Wang Z. Tang Q. (2019). Phytochemical profile and antidepressant effect of *Ormosia henryi* Prain leaf ethanol extract. Int. J. Mol. Sci. 20, 3396. 10.3390/ijms20143396 31295954 PMC6678957

[B30] Lueptow L. M. (2017). Novel object recognition test for the investigation of learning and memory in mice. J. Vis. Exp., 30 (126), 55718. 10.3791/55718 28892027 PMC5614391

[B31] McGirr S. Venegas C. Swaminathan A. (2020). Alzheimers disease: a brief review. J. Exp. Neurology 1, 89–98. 10.33696/Neurol.1.015

[B32] Moore S. J. Deshpande K. Stinnett G. S. Seasholtz A. F. Murphy G. G. (2013). Conversion of short-term to long-term memory in the novel object recognition paradigm. Neurobiol. Learning Memory 105, 174–185. 10.1016/j.nlm.2013.06.014 23835143 PMC3786371

[B33] Morales-Delgado N. Popović N. De la Cruz-Sánchez E. Caballero Bleda M. Popović M. (2018). Time-of-day and age impact on memory in elevated plus-maze test in rats. Front. Behavioral Neuroscience 12, 304. 10.3389/fnbeh.2018.00304 PMC6291441 30574075

[B34] Mostafa N. M. Abd El-Ghffar E. A. Hegazy H. G. Eldahshan O. A. (2018). New methoxyflavone from Casimiroa sapota and the biological activities of its leaves extract against lead acetate induced hepatotoxicity in rats. ChemistryBiodiversity 15, e1700528. 10.1002/cbdv.201700528 29411525

[B35] Otari K. V. Bichewar O. G. Shete R. V. Upasani C. D. (2012). Effect of hydroalcoholic extract of *Vitex negundo* Linn. leaves on learning and memory in normal and cognitive deficit mice. Asian Pac. J. Trop. Biomed. 2, S104–S111. 10.1016/S2221-1691(12)60138-5

[B36] Prinz S. Ringl A. Huefner A. Pemp E. Kopp B. (2007). 4′′′‐Acetylvitexin‐2 ″‐*O*‐rhamnoside, isoorientin, orientin, and 8‐methoxykaempherol‐3‐O‐glucoside as markers for the differentiation of *Crataegus monogyna* and *Crataegus pentagyna* from *Crataegus laevigata* (Rosaceae). ChemistryBiodiversity 4, 2920–2931. 10.1002/cbdv.200790241 18081102

[B37] Promprom W. Chatan W. Munglue P. (2020). Effect of *Vitex pinnata* L. leaf extract on estrogenic activity and lipid profile in ovariectomized rats. Pharmacogn. Mag. 16, S492–S497. 10.4103/pm.pm_443_19

[B38] Qiu C. Kivipelto M. Von Strauss E. (2009). Epidemiology of Alzheimer's disease: occurrence, determinants, and strategies toward intervention. Dialogues Clinical Neuroscience 11, 111–128. 10.31887/DCNS.2009.11.2/cqiu 19585947 PMC3181909

[B39] Rahiman R. A. Rajan N. Sreekumaran E. (2015). Neuroprotective effect of vitex negundo against scopolamine induced cognitive impairment and oxidative stress in Wistar albino rats. Biosci. Biotechnol. Res. Asia 12, 301–307. 10.13005/bbra/2204

[B40] Rajesh V. Riju T. Venkatesh S. Babu G. (2017). Memory enhancing activity of *Lawsonia inermis* Linn. leaves against scopolamine induced memory impairment in Swiss albino mice. Orient. Pharm. Exp. Med. 17, 127–142. 10.1007/s13596-017-0268-8

[B41] Saeed AL-Wajeeh N. Halabi M. Hajrezaie Dhiyaaldeen S. Bardi D. Salama S. (2016). The gastroprotective effect of *Vitex pubescens* leaf extract against ethanol-provoked gastric mucosal damage in sprague-dawley rats. PloS One 11, e0157431. 10.1371/journal.pone.0157431 27689880 PMC5045201

[B42] Scott K. N. (1972). Carbon-13 nuclear magnetic resonance of biologically important aromatic acids. I. Chemical shifts of benzoic acid and derivatives. J. Am. Chem. Soc. 94, 8564–8568. 10.1021/ja00779a045 4638987

[B43] Sharma A. C. Kulkarni S. K. (1992). Evaluation of learning and memory mechanisms employing elevated plus-maze in rats and mice. Prog. Neuro-Psychopharmacology Biol. Psychiatry 16, 117–125. 10.1016/0278-5846(92)90014-6 1557503

[B44] Sun Y. Tsao R. Chen F. Li H. Wang J. Peng H. (2017). The phytochemical composition, metabolites, bioavailability and *in vivo* antioxidant activity of *Tetrastigma hemsleyanum* leaves in rats. J. Funct. Foods 30, 179–193. 10.1016/j.jff.2017.01.004

[B45] Thenmozhi S. Subasini U. (2016). *In vitro* cytotoxic activity of various fractions of hydroalcoholic extract of *Vitex pinnata* linn leaves against EAC cell lines. World Journal Pharmacy Pharmaceutical Sciences, 5 (6). 10.20959/wjpps20166-7038

[B46] Tian T. Zeng J. Zhao G. Zhao W. Gao S. Liu L. (2018). Neuroprotective effects of orientin on oxygen-glucose deprivation/reperfusion-induced cell injury in primary culture of rat cortical neurons. Exp. Biology Medicine 243, 78–86. 10.1177/1535370217737983 29073777 PMC5788155

[B47] Tijjani A. Ndukwe I. Ayo R. (2012). Isolation and characterization of lup-20 (29)-ene-3, 28-diol (Betulin) from the stem-bark of *Adenium obesum* (Apocynaceae). Trop. J. Pharm. Res. 11, 259–262. 10.4314/tjpr.v11i2.12

[B48] Widmann C. N. Beinhoff U. Riepe M. (2012). Everyday memory deficits in very mild Alzheimer's disease. Neurobiol. Aging 33, 297–303. 10.1016/j.neurobiolaging.2010.03.012 20392540

[B49] Youssef F. S. Labib R. M. Eldahshan O. A. Singab A. N. B. (2017). Synergistic hepatoprotective and antioxidant effect of artichoke, fig, blackberry herbal mixture on HepG2 cells and their metabolic profiling using NMR coupled with chemometrics. Chemistry& Biodivers. 14, e1700206. 10.1002/cbdv.201700206 28898531

[B50] Zhang Z. Liao L. Moore J. Wu T. Wang Z. (2009). Antioxidant phenolic compounds from walnut kernels (*Juglans regia* L.). Food Chemistry 113, 160–165. 10.1016/j.foodchem.2008.07.061

[B51] Zhong Y. Zheng Q. Y. Sun C. Y. Zhang Z. Han K. Jia N. (2019). Orientin improves cognition by enhancing autophagosome clearance in an Alzheimer’s mouse model. J. Mol. Neurosci. 69, 246–253. 10.1007/s12031-019-01353-5 31243684

[B52] Zlotnik G. Vansintjan A. (2019). Memory: an extended definition. Front. Psychology 10, 2523. 10.3389/fpsyg.2019.02523 31787916 PMC6853990

