# Supplementary material for: Neuroprotective effect of standardized Vitex pubescens Vahl bark extract and its n-butanol fraction against scopolamine-induced cognitive impairment in Sprague-Dawley rats in relationship to its isolated phytochemicals
Source: Front Pharmacol. 2026 Apr 17;17:1775571. doi: 10.3389/fphar.2026.1775571 (PMC13132829; doi:10.3389/fphar.2026.1775571)
Supplement: Supplementary file 1 [file Supplementaryfile1.docx]

**SUPPLEMENTARY MATERIAL**

**Neuroprotective effect of standardized *Vitex pubescens* Vahl bark extract and its *n*-butanol fraction against scopolamine-induced cognitive impairment in Sprague-Dawley rats in relationship to its isolated phytochemicals**

**
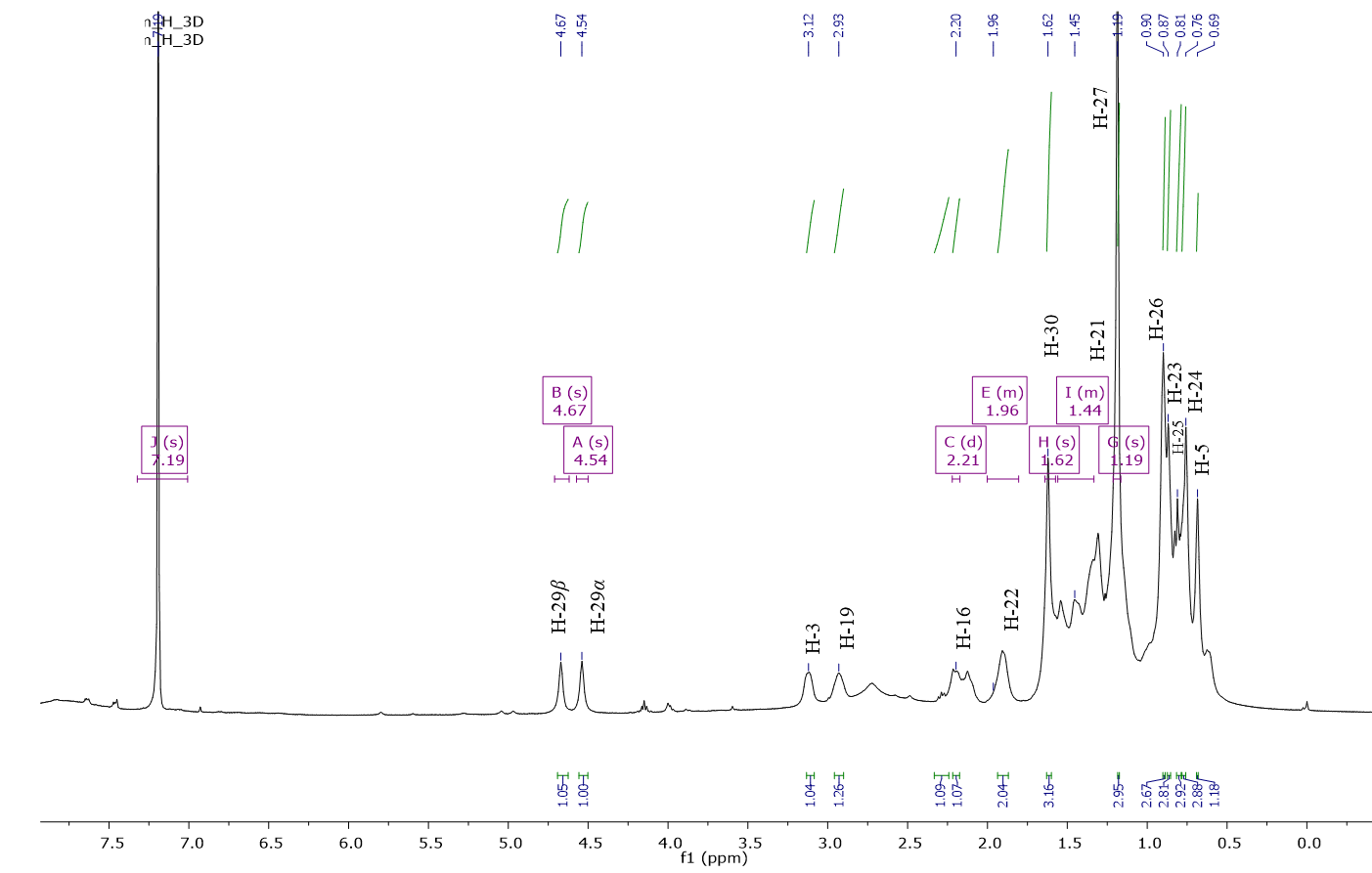

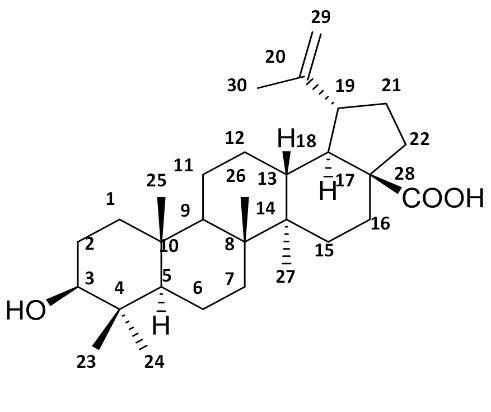
**

**Figure S1.** ^1^ H-NMR spectrum of compound **(V1)**Betulinic acid


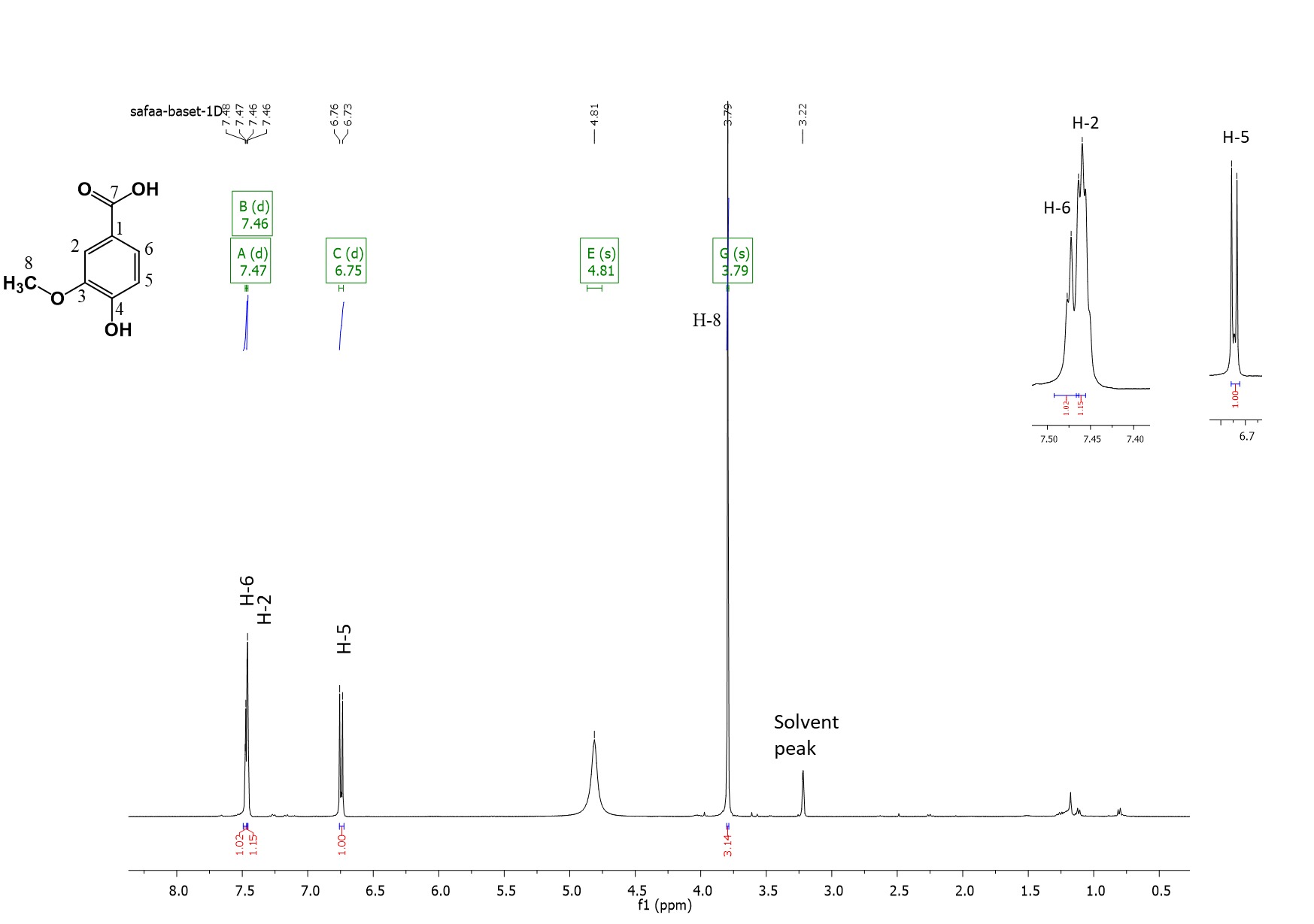


**Figure S2.** ^1^ H-NMR spectrum of compound **(V2)**

Vanillic acid

C-7

C-4

C-3

C-6

C-1

C-2

C-5

C-8

8

6

5

4

3

2

1

7

**Figure S3.** ^13^ C-NMR spectrum of compound **(V2)**

Vanillic acid

Solvent peak

**Figure S4.** ^1^ H-NMR spectrum of compound **(V3)**

*P*-hydroxy benzoic acid


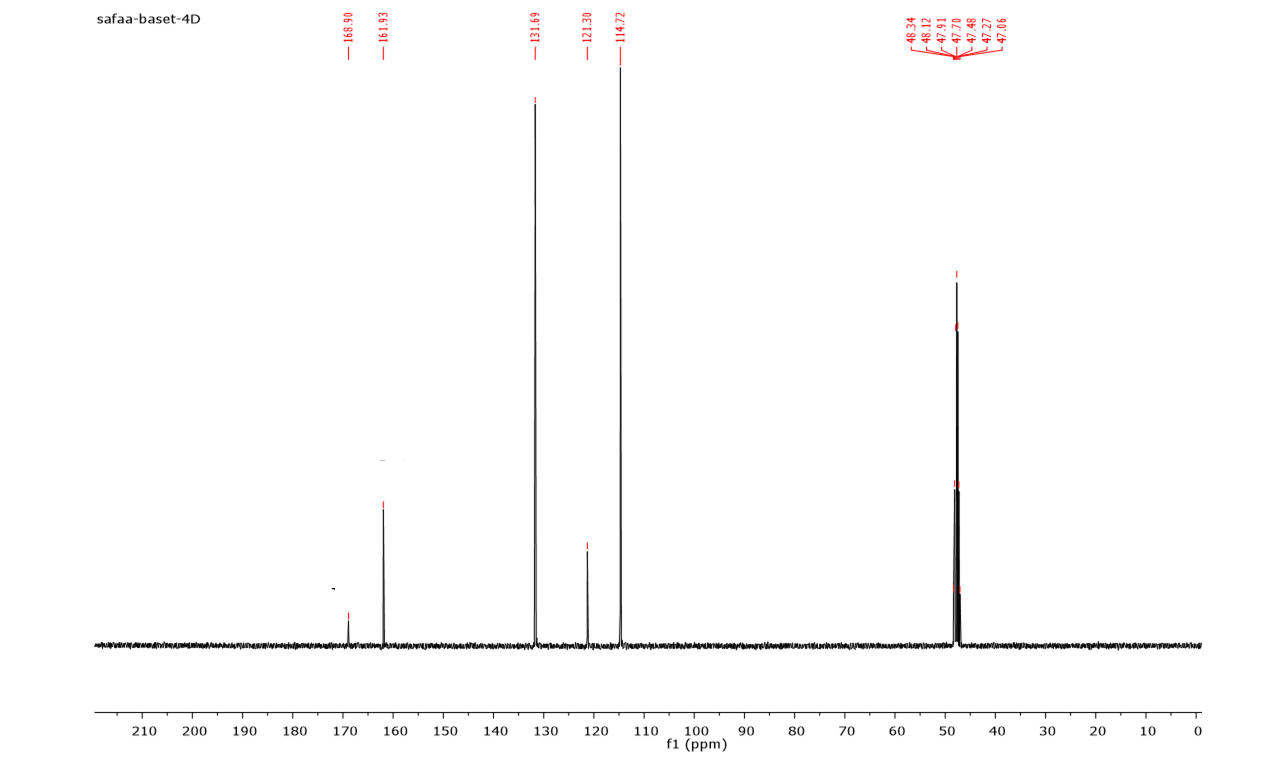


C-7

C-4

C-1

C-2,6

C-3,5

**Figure S5.** ^13^ C-NMR spectrum of compound **(V3)**

*P*-hydroxy benzoic acid


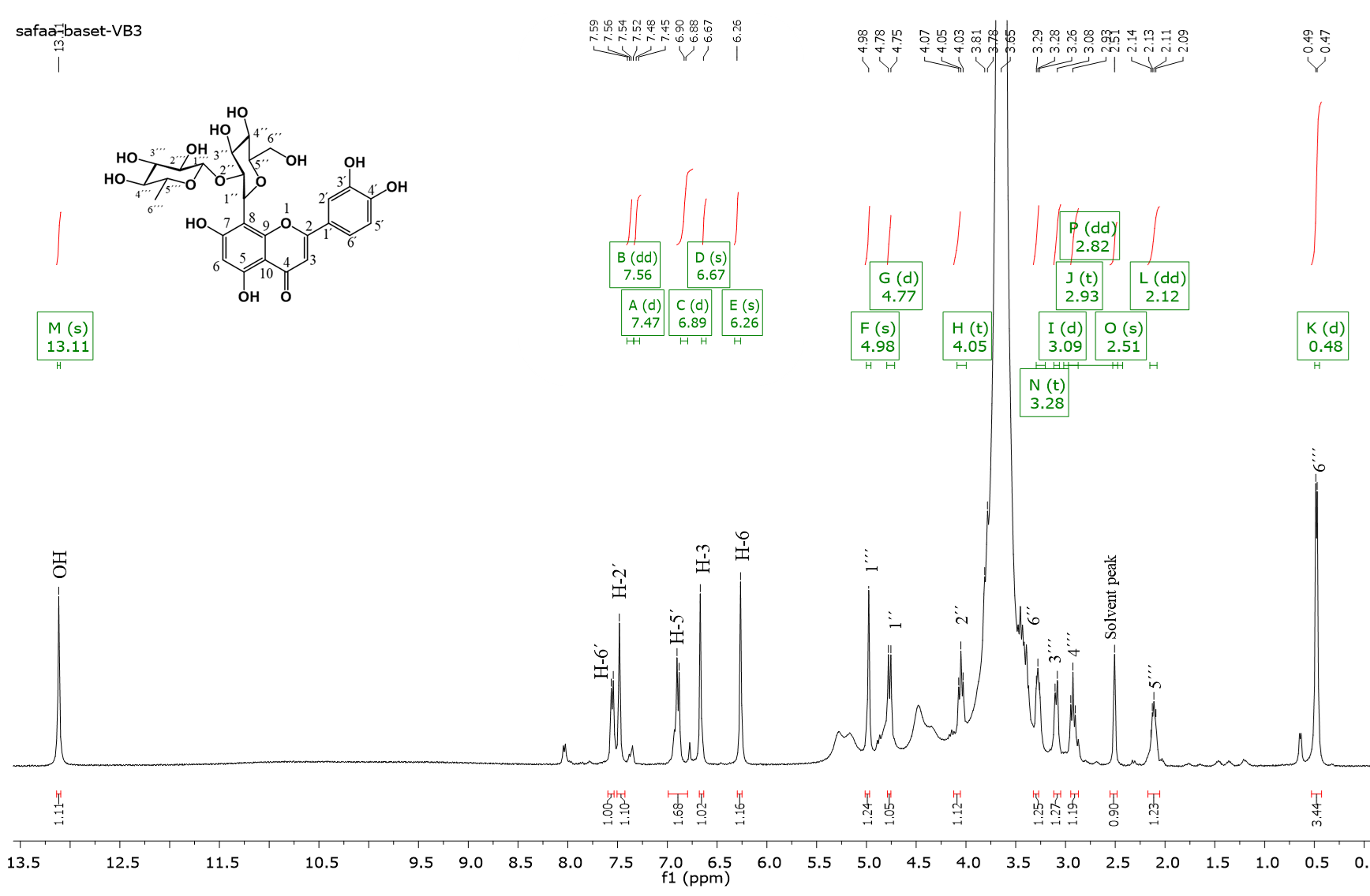


**Figure S6.** ^1^ H-NMR spectrum of compound **(V4)**

Orientin-2´ˊ-*O*-*α*-L-rhamnoside


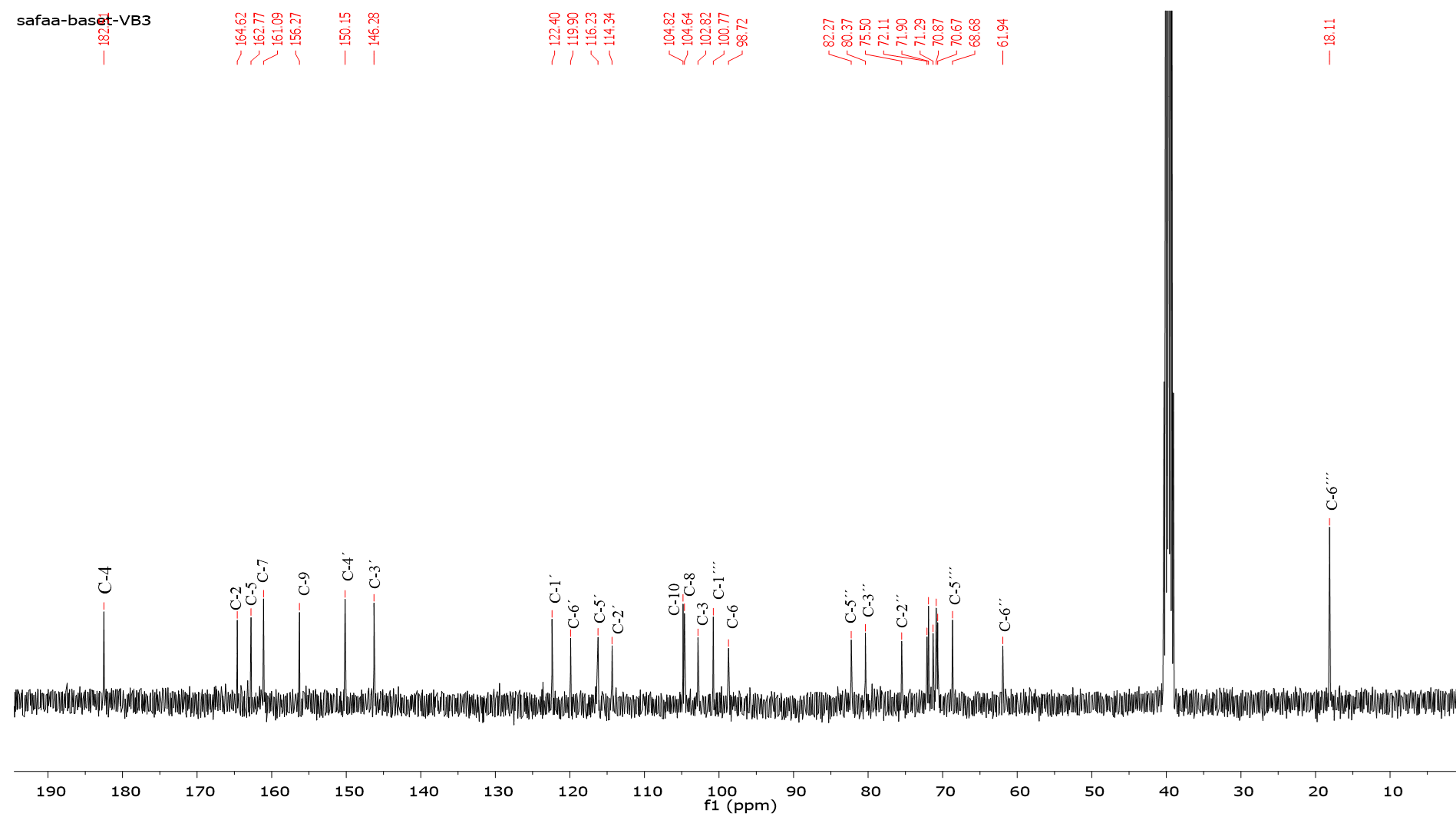

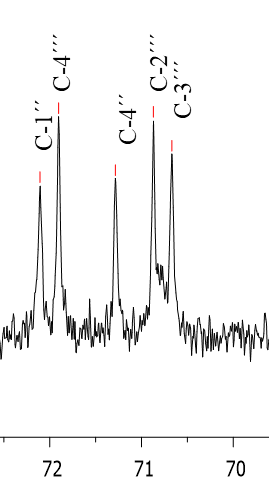

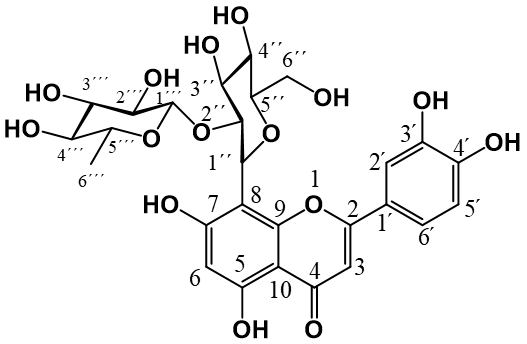


**Figure S7.** ^13^ C-NMR spectrum of compound **(V4)**

Orientin-2´ˊ-*O*-*α*-L-rhamnoside

**
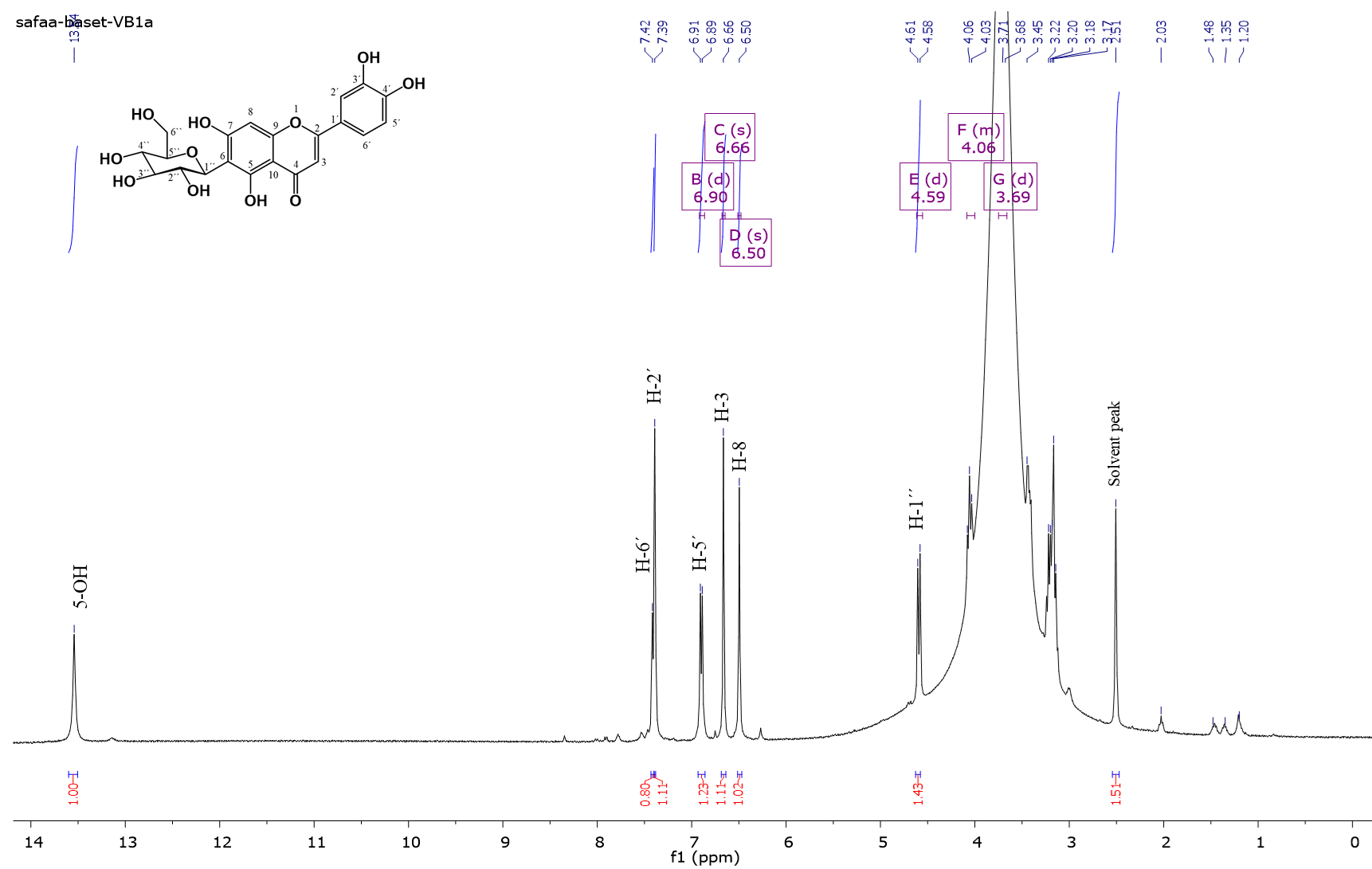
**

**Figure S8.** ^1^ H-NMR spectrum of compound **(V5)**

Isoorientin

**
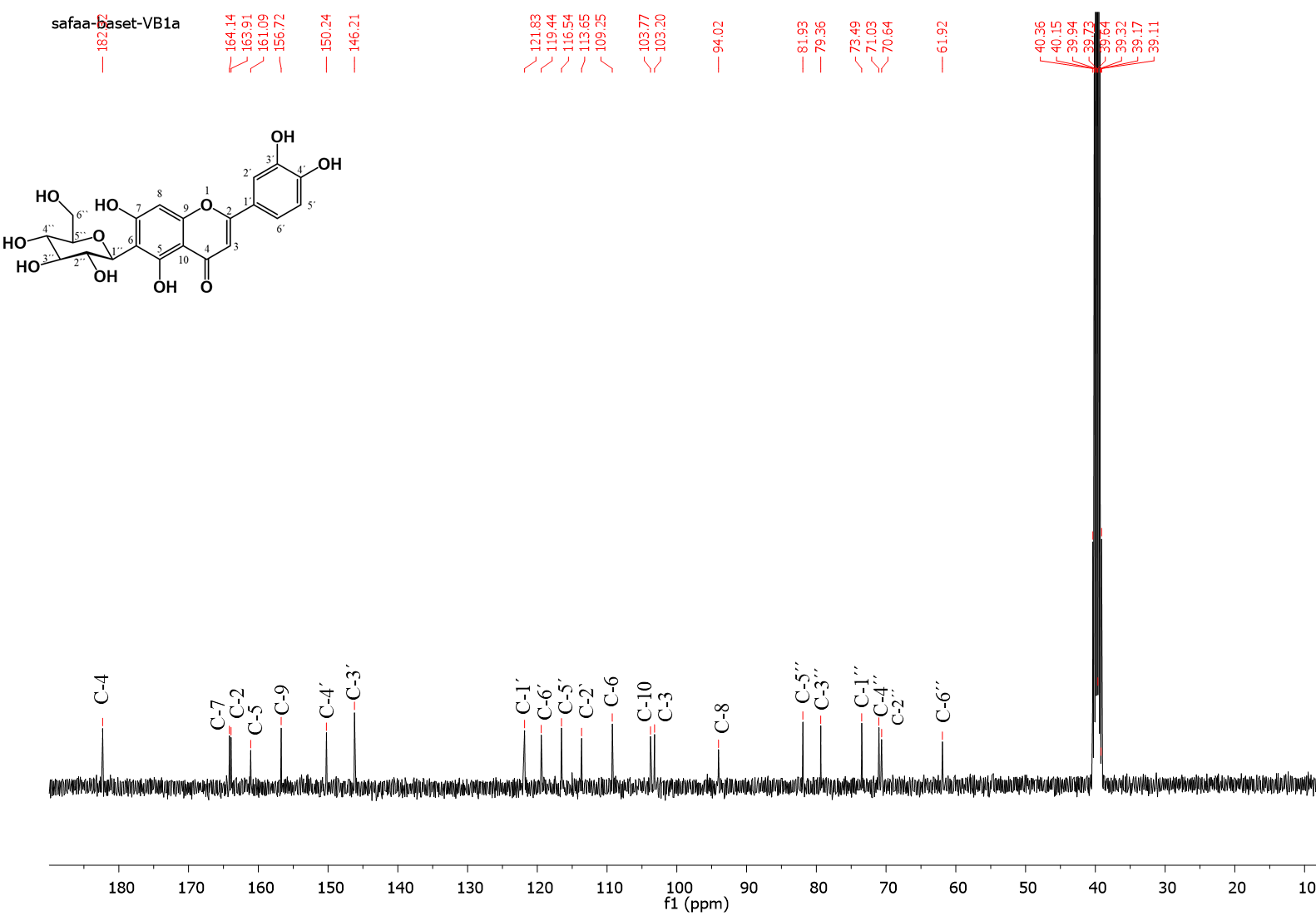
**

**Figure S9.** ^13^ C-NMR spectrum of compound **(V5)**

Isoorientin


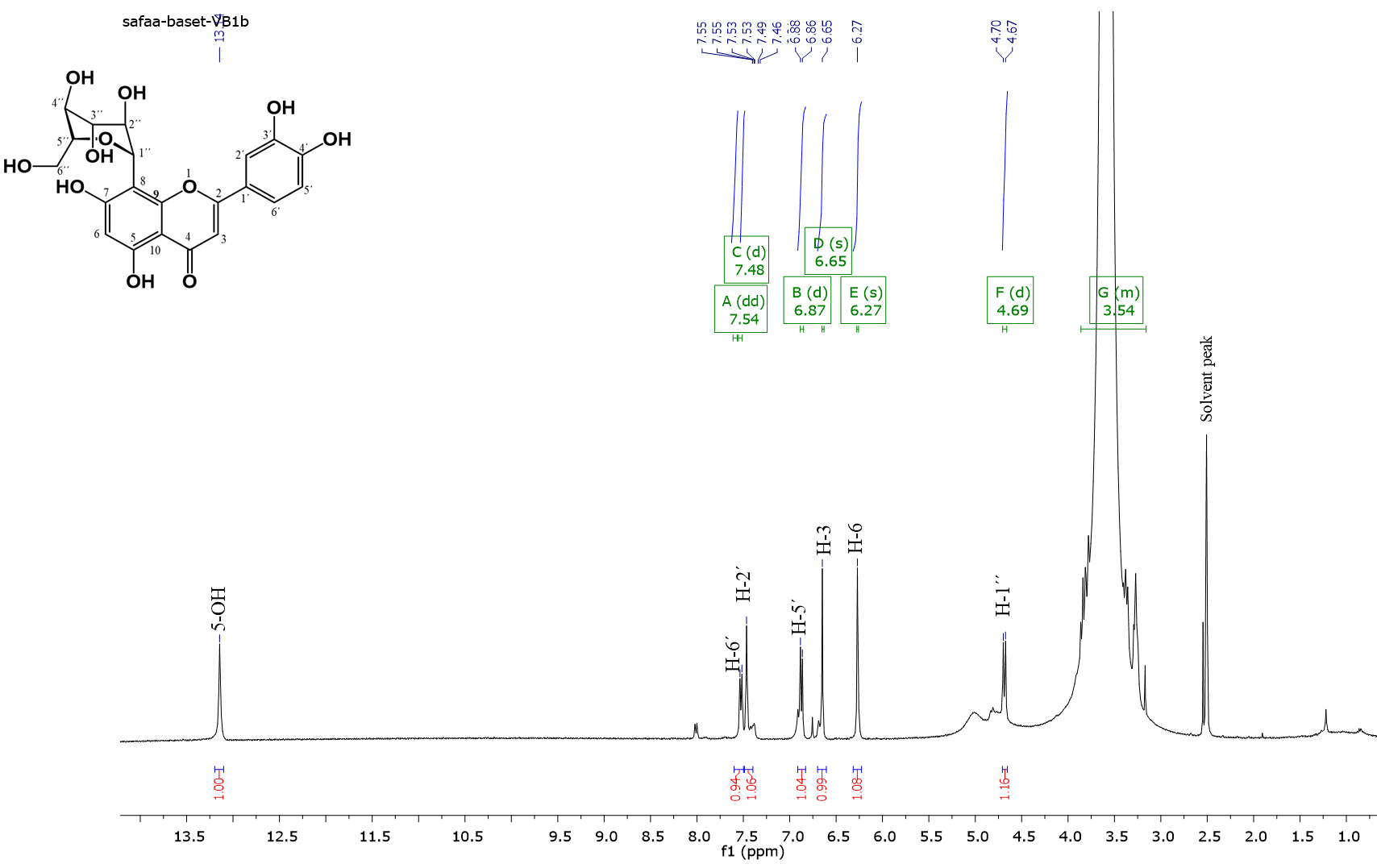


**Figure S10.** ^1^ H-NMR spectrum of compound **(V6)**

Orientin


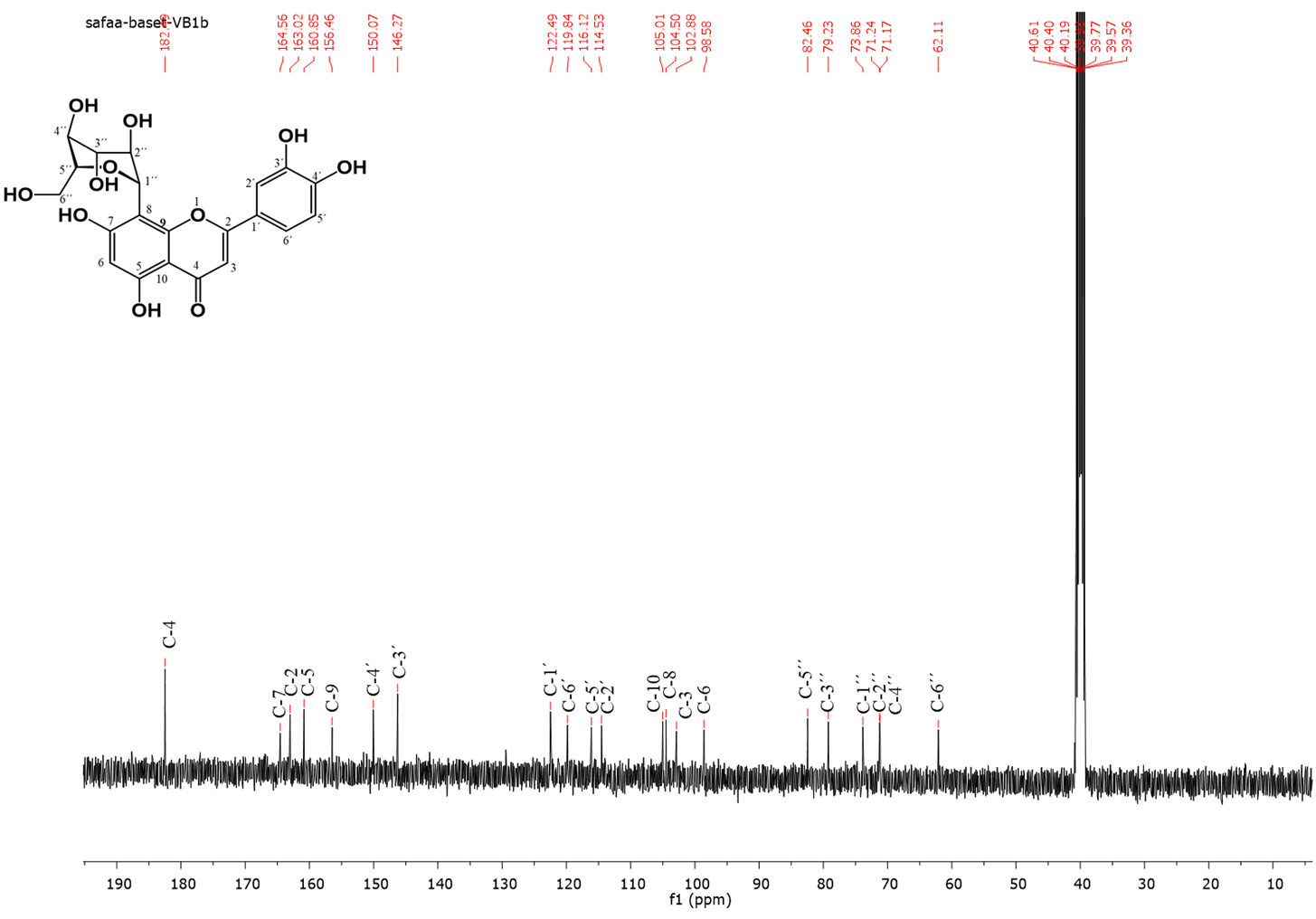


**Figure S11.** ^13^C-NMR spectrum of compound **(V6)**

Orientin
